# Multiparametric ECIS Profiling Complements Metabolic Endpoint Screening of Complex Plant Extracts in HNSCC Models

**DOI:** 10.3390/bios16100563

**Published:** 2026-10-05

**Authors:** Fine Handrick, Vincent O. Imieje, Vivien Engel, Esther Abiodun Odigie, Ewelukwa Ebube Chukwueloka, Lilian Nneoma Amafili, Onome Keturah Evi, Nadja Engel

**Affiliations:** 1 Department of Oral, Maxillofacial and Plastic Surgery, Rostock University Medical Center, Schillingallee 35, 18057 Rostock, Germany; fine_m@outlook.de (F.H.); vivien.engel@med.uni-rostock.de (V.E.); 2 Department of Pharmaceutical Chemistry, Faculty of Pharmacy, University of Benin, Ugbowo, Benin City 300001, Nigeria; vincent.imieje@uniben.edu (V.O.I.); esther.odigie@pharm.uniben.edu (E.A.O.); ebube.ewelukwa@pharm.uniben.edu (E.E.C.); nneoma.amafili@pharm.uniben.edu (L.N.A.); onome.evi@pharm.uniben.edu (O.K.E.); 3 Oscar Langendorff Institute of Physiology, Rostock University Medical Center, Gertrudenstrasse 9, 18057 Rostock, Germany

**Keywords:** cell-based biosensor, electric cell–substrate impedance sensing, head and neck squamous cell carcinoma, phenotypic screening, plant extracts, barrier resistance, live-cell monitoring

## Abstract

Metabolic endpoint assays enable rapid screening but provide limited information on treatment kinetics, heterogeneity, and recovery. Here, we established a multistep workflow combining MTS-based metabolic screening with electric cell–substrate impedance sensing (ECIS), a label-free cell-based biosensor, to functionally prioritize complex plant extracts in head and neck squamous cell carcinoma (HNSCC) models. Seventeen soluble extracts were screened at 50 µg/mL for 24 h in four HNSCC cell lines and human adipose-derived stem cells. Selected candidates were characterized by real-time impedance monitoring, multifrequency analysis, and model-derived barrier resistance. Extract 16 showed the most favorable metabolic selectivity profile. ECIS resolved no growth-inhibitory effects, sustained impedance suppression, heterogeneous responses, and transient suppression followed by partial recovery. Recovery was incomplete (CAL-33) or absent (Detroit 562); modeled barrier resistance became resolvable late (FaDu, PE/CA-PJ15) or not at all (CAL-33). Comparison with the PI3Kα inhibitor Inavolisib revealed partially overlapping but non-identical metabolic response profiles and no consistent enhancement by combination treatment. Extract 16 was associated with junctional redistribution, F-actin remodeling, and cell line-dependent PARP cleavage, whereas caspase 3/7 activation could not be detected. Integrating metabolic endpoint screening with multiparametric ECIS monitoring provides greater functional resolution and supports prioritizing complex bioactive samples for chemical and mechanistic investigation.

## 1. Introduction

Head and neck squamous cell carcinoma (HNSCC) accounts for the majority of malignancies arising in the oral cavity, pharynx, and larynx and continues to represent a substantial global disease burden [1,2]. Despite progress in surgery, radiotherapy, systemic treatment, and immunotherapy, recurrence, treatment resistance, and metastatic progression remain major clinical challenges. HNSCC comprises a biologically heterogeneous group of tumors whose molecular characteristics and treatment responses vary by anatomical site, etiological background, and human papillomavirus status [2,3]. Genomic studies have identified recurrent alterations in pathways controlling cell-cycle progression, epithelial differentiation, growth-factor signaling, and cellular survival. Among these, aberrations affecting the PI3K/AKT/mTOR axis are frequently observed in HNSCC, including activating *PIK3CA* variants [3,4]. However, molecular alterations do not necessarily predict a uniform functional response to pathway-directed treatment, and only a limited number of proposed predictive biomarkers have been sufficiently validated for clinical or experimental use [5]. Functional cellular assays can therefore complement molecular annotation by directly resolving treatment responses across different tumor models. Natural products and plant-derived extracts are important sources of structurally diverse bioactive compounds and have contributed substantially to anticancer drug discovery [6]. Their chemical complexity and potential polypharmacology make them suitable for phenotypic screening strategies that do not rely on predefined molecular targets [7]. At the same time, complex extracts pose methodological challenges, including limited solubility, variability in composition, assay interference, and the subsequent identification of active constituents. A staged functional workflow may therefore help prioritize extracts or fractions for more detailed chemical and mechanistic characterization.

Colorimetric tetrazolium assays such as MTS are widely used for initial compound screening because they are technically straightforward, scalable, and compatible with multiwell formats. However, MTS conversion reflects cellular reductive metabolism rather than cell number, proliferation, or cell death directly [8]. Metabolic adaptation can therefore generate signals that do not correlate linearly with cellular abundance. Moreover, static endpoint assays provide no information on the onset, persistence, heterogeneity, or reversibility of a response. Electric cell–substrate impedance sensing (ECIS) provides continuous, label-free monitoring of adherent cellular systems [9,10]. Impedance changes integrate several aspects of cell behavior, including electrode coverage, cell morphology, cell–substrate adhesion, and cell–cell interactions. Multifrequency measurements and model-derived parameters can provide additional biophysical information beyond the global impedance signal [11,12,13]. ECIS is therefore well suited to complement endpoint assays by resolving dynamic response profiles and recovery behavior, particularly when chemically complex samples are investigated [14].

In the present study, we established a multistep functional screening workflow combining MTS-based metabolic screening with real-time ECIS profiling in four HNSCC cell lines and human adipose-derived stem cells as a non-malignant comparator. Extracts were initially ranked based on their metabolic inhibition and tumor-selectivity profiles. Selected candidates were subsequently characterized by real-time impedance monitoring, multifrequency analysis, and model-derived barrier-resistance assessment. The novelty of this work is methodological. It does not lie in a single compound but in linking tumor/non-malignant metabolic screening with continuous impedance recording across a cell-line panel, including a washout phase and quantitative, control-referenced metrics of response magnitude, persistence, and recovery. Because endpoint assays such as MTS remain the standard readout in extract and compound screening, we compared them directly with continuous impedance monitoring on the same extracts and cell lines to show which response information a single endpoint misses. We hypothesized that integrating metabolic endpoint measurements with dynamic and biophysical ECIS readouts would reveal treatment-response phenotypes that remain unresolved by conventional screening alone and thereby improve the functional prioritization of complex bioactive samples.

## 2. Materials and Methods

### 2.1. Plant Material, Authentication, and Extract Preparation

Plant materials were purchased from the Ikpoba Hill Spice Market in Benin City, Nigeria, on 19 January 2023. Botanical authentication was performed by Prof. Aigbokhan Emmanuel Izaka at the Herbarium Unit, Department of Plant Biology and Biotechnology, Faculty of Life Sciences, University of Benin, Benin City, Nigeria. Voucher specimens were deposited at the herbarium, and the corresponding voucher numbers are listed in Table 1. The seed material corresponding to internal sample codes CA-H-07 and CA-D-07 was subsequently re-authenticated as *Ricinodendron heudelotii* (Baill.) Pierre ex Heckel; voucher specimen UBH-R623 was retained, and the internal sample codes and extraction assignments remained unchanged. Dried seeds, fruits, or root bark were ground to a coarse powder using a mechanical blender at the Department of Pharmacognosy, Faculty of Pharmacy, University of Benin. Depending on the available plant material, 200–600 g of powdered material were transferred to maceration vessels and successively extracted with 350–700 mL of n-hexane, dichloromethane, and/or methanol for 72 h. All solvents were of analytical grade (Thermo Fisher Scientific, Waltham, MA, USA). Extracts were filtered through Whatman No. 1 filter paper (Cytiva, Marlborough, MA, USA) and concentrated under reduced pressure using a rotary evaporator at 45 °C. The resulting extracts were weighed, assigned individual codes, transferred to airtight containers, and stored under refrigerated conditions until use. For cell-based experiments, all plant extracts were prepared as 50 mg/mL stock solutions in dimethyl sulfoxide (DMSO; VWR/BDH Prolabo, Darmstadt, Germany). Working solutions were prepared by a 1:1000 dilution of the respective stock solution into assay medium, resulting in a final extract concentration of 50 µg/mL and a final DMSO concentration of 0.1% (*v*/*v*). Extract 16 corresponded to the dichloromethane seed extract assigned internal sample code CA-D-07. Extracts 9, 11, 17, and 18 were excluded from biological screening because suitable assay solutions could not be prepared owing to insufficient solubility.

### 2.2. Cell Lines, Culture Conditions, and Molecular Annotation

Four human HNSCC cell lines were used: CAL-33 (ACC 447; Leibniz Institute DSMZ, Braunschweig, Germany), FaDu (ACC 784; Leibniz Institute DSMZ), PE/CA-PJ15 (ECACC 96121230; European Collection of Authenticated Cell Cultures, Salisbury, UK), and Detroit 562 (CCL-138; American Type Culture Collection, Manassas, VA, USA). CAL-33 and PE/CA-PJ15 were derived from squamous cell carcinomas of the oral tongue, FaDu from a hypopharyngeal squamous cell carcinoma, and Detroit 562 from a metastatic pleural effusion associated with pharyngeal squamous cell carcinoma. Selected molecular characteristics were compiled from Cellosaurus and the published literature and summarized in Table 2 [4,15,16,17,18]. These annotations were used exclusively to provide molecular context and were not independently determined in the present study.

HNSCC cells were maintained as adherent monolayer cultures in Dulbecco’s modified Eagle’s medium containing UltraGlutamine (Lonza, Verviers, Belgium), supplemented with 10% fetal calf serum (PAN-Biotech, Aidenbach, Germany) and 1% antibiotic–antimycotic solution (Gibco, Paisley, UK). Cells were cultured at 37 °C in a humidified atmosphere containing 5% CO_2_ (Binder GmbH, Tuttlingen, Germany) and passaged with 0.05% trypsin/0.02% EDTA (Thermo Fisher Scientific) before reaching complete confluence. Human adipose-derived stem cells (hASCs) were used as a non-malignant mesenchymal comparator. They have long been established in our laboratory, are cultured at defined passage numbers, and have been characterized at the molecular level. They grow as adherent monolayers on ECIS electrodes and could be analyzed with the same MTS and ECIS protocols as the tumor cell lines. Because tissue lost during head and neck tumor resection has to be reconstructed and adipose-derived stem cells are of interest for this regenerative purpose, hASCs also served to assess whether candidate extracts impair a non-malignant cell population relevant to regeneration. hASCs were isolated from lipoaspirate obtained from patients undergoing liposuction or lipofilling procedures at Rostock University Medical Center as previously described [19]. The MTS screen was performed with hASCs from donor Z06/22 (female, 33 years; passages 5–6) in two independent experiments; a third screening experiment with hASCs from a second donor was not included because these cells proliferated poorly and stopped proliferating in the following passage. The ECIS experiment and the concentration–response experiments were performed with hASCs from donor Z06/22. Sample collection and cell isolation were approved by the Ethics Committee of Rostock University Medical Center (approval no. A 2014-0092), and written informed consent was obtained from all donors. hASCs were expanded in MSC growth medium (PromoCell, Heidelberg, Germany). For the experiments, hASCs were maintained in a medium consisting of 45% Iscove’s Modified Dulbecco’s Medium, 45% F-12 Nutrient Mixture, and 10% newborn calf serum, supplemented with penicillin–streptomycin and recombinant human basic fibroblast growth factor at 10 ng/mL (Gibco, Schwerte, Germany, for Iscove’s Modified Dulbecco’s Medium and F-12 Nutrient Mixture; Thermo Fisher Scientific, for all other components). Cells were cultured at 37 °C in a humidified atmosphere containing 5% CO_2_.

### 2.3. Primary MTS-Based Extract Screening

**Figure 1:**
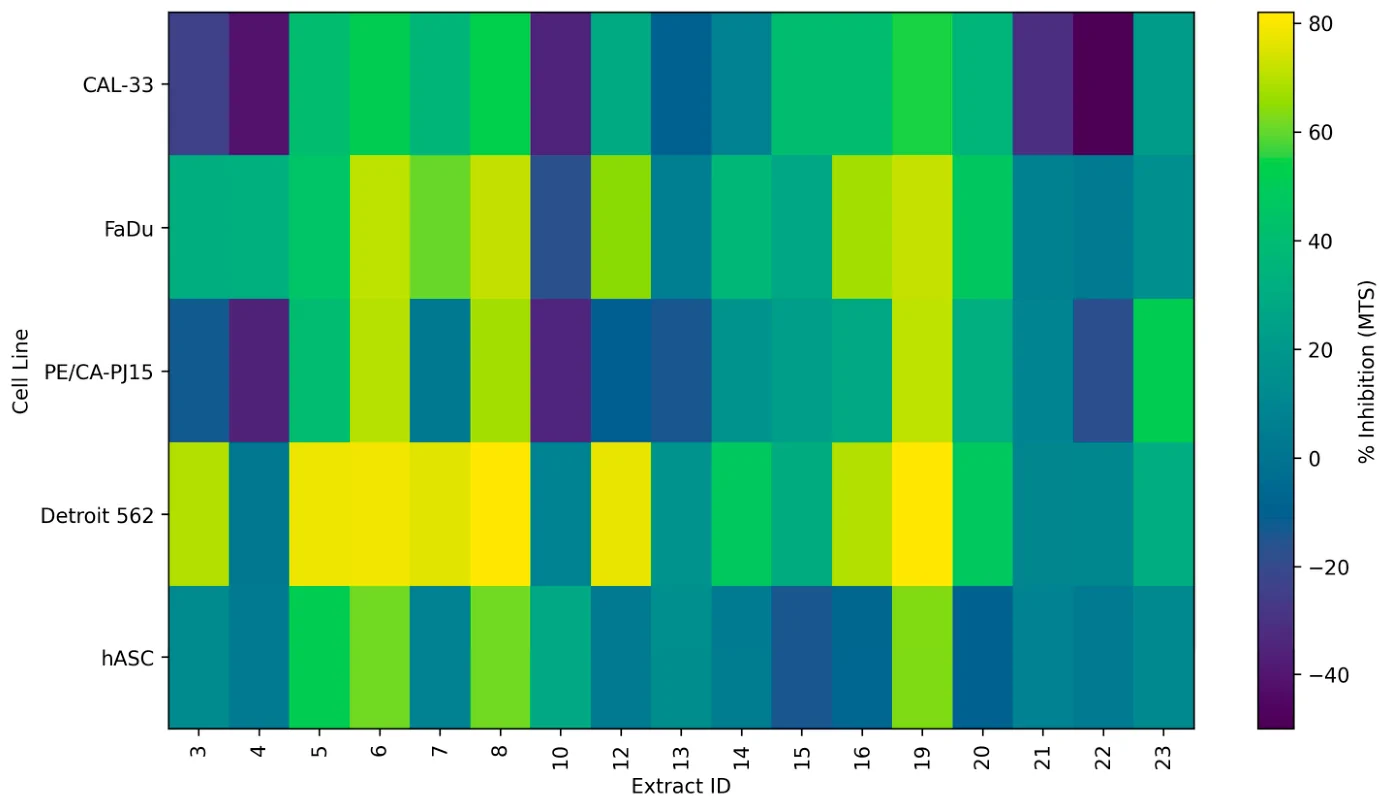
MTS-based metabolic inhibition profiles of plant extracts across HNSCC cell lines and hASCs. Heatmap showing percent inhibition, calculated as 100 − metabolic activity (%), after treatment with the indicated plant extracts at 50 µg/mL for 24 h in CAL-33, FaDu, PE/CA-PJ15, and Detroit 562 HNSCC cells and in human adipose-derived stem cells (hASCs). *Metabolic activity* was normalized to the respective DMSO control, which was set to 100% metabolic activity. Values are from one representative experiment of three (CAL-33, FaDu, PE/CA-PJ15, and Detroit 562) or two (hASC) independent biological experiments; all experiments are shown as mean ± SD in Supplementary Figure S1. Positive values indicate reduced metabolic activity relative to the DMSO control, whereas negative values indicate metabolic activity above the respective control level. The color scale ranges from low or negative inhibition to pronounced metabolic inhibition.

The MTS-based screening workflow was adapted from previously established plant-extract screening approaches of our group [20,21]. Cells were seeded into 96-well plates (Sarstedt AG & Co. KG, Nümbrecht, Germany) at 10,000 cells per well in their respective growth media. After 24 h, cells were washed once with phosphate-buffered saline (PBS) (Merck KGaA, Darmstadt, Germany), and the culture medium was replaced with phenol-red-free DMEM (Gibco, Schwerte, Germany) supplemented with 10% charcoal-stripped fetal bovine serum (PAN-Biotech) and 1% penicillin–streptomycin. Cells were incubated for a further 24 h before treatment. Plant extracts were diluted in assay medium to a final nominal concentration of 50 µg/mL. The final DMSO concentration was maintained at 0.1% (*v*/*v*) in all treatment and vehicle-control wells. Cells were exposed to the extracts for 24 h. DMSO-treated cells served as the vehicle control. Cell-free wells containing the corresponding treatment solutions were included for background correction and to control for extract-associated assay interference. *Metabolic activity* was determined using the CellTiter 96^®^ AQueous One Solution Cell Proliferation Assay (MTS; Promega, Madison, WI, USA) according to the manufacturer’s instructions. Ten microliters of MTS reagent were added per well, and plates were incubated for 30–60 min at 37 °C. Absorbance was measured at 492 nm using an Absorbance 96 plate reader (Byonoy GmbH, Hamburg, Germany). The primary extract screening was performed in three independent biological experiments for CAL-33, FaDu, PE/CA-PJ15, and Detroit 562, and in two independent biological experiments for hASCs. Figure 1 shows one representative experiment; all experiments are shown as mean ± SD in Supplementary Figure S1.

*Metabolic activity* (%) was calculated relative to the DMSO control:

$$Metabolic\;activity\;(\%)\;=\;\frac{\left(A_{treated}\;-\;A_{blank}\right)}{\left(A_{DMSO}\;-\;A_{blank}\right)}\;\times \;100$$

*Metabolic inhibition* was calculated as follows:

Metabolic inhibition (%) = 100 − Metabolic activity (%)

Negative inhibition values therefore indicate metabolic activity above the respective DMSO-control level. For each extract, mean tumor inhibition was calculated as the arithmetic mean of the inhibition values obtained in CAL-33, FaDu, PE/CA-PJ15, and Detroit 562 cells. Tumor selectivity was visualized by plotting mean tumor inhibition against hASC metabolic activity. The extended selectivity zone was defined as mean tumor inhibition of at least 25% combined with hASC metabolic activity of at least 90% of the DMSO control. The strict selectivity zone was defined as mean tumor inhibition of at least 50% combined with hASC metabolic activity of at least 100% of the DMSO control.

### 2.4. Electric Cell–Substrate Impedance Sensing

ECIS measurements and multifrequency analysis were performed according to previously established impedance-sensing workflows of our group, with adaptations for the present extract-screening design [20,22]. Electric cell–substrate impedance sensing experiments were performed using an ECIS Z-Theta 96-Well Array Station and 96W20idf PET arrays containing interdigitated gold electrodes (Applied BioPhysics, Troy, NY, USA). The ECIS station was equilibrated in a humidified incubator at 37 °C and 5% CO_2_ for 24 h before each experiment. The electrode arrays were pre-equilibrated with 200 µL serum-free DMEM(Gibco, Schwerte, Germany) per well for the corresponding baseline period. Impedance (*Z*), resistance (*R*), and capacitance (*C*) were recorded at seven frequencies: 4, 12, 16, 24, 32, 48, and 64 kHz. Data were acquired with ECIS software version 1.4.12.0 and exported and modelled with version 1.4.18.0 (Applied BioPhysics). Following the baseline measurement, 10,000 cells were seeded per well in 200 µL of the appropriate cell-culture medium. Selected plant extracts were added at a final concentration of 50 µg/mL, corresponding to a 1:1000 dilution of the 50 mg/mL DMSO stock solution and a final DMSO concentration of 0.1% (*v*/*v*). DMSO-treated cells served as the vehicle control. Cell-free wells containing culture medium but no cells were included to monitor electrode- and medium-associated background signals. Untreated cells were included where indicated. After completion of the treatment phase, the treatment-containing medium was replaced with extract-free culture medium, and impedance monitoring was continued to assess post-treatment recovery. Technical replicate numbers differed between individual extracts and experimental plates and are specified in the corresponding figure legends.

#### 2.4.1. Plate-Specific ECIS Phases and Impedance Normalization

Because the cell lines were analyzed on separate ECIS plates, the exact phase transitions differed between experiments. The following plate-specific time intervals were used (Table 3):

For the primary dynamic analysis, impedance at 16 kHz was normalized separately for each well to the measurement obtained at the respective cell-seeding time point:

$$Z_{norm}(t)\;=\;\frac{Z(t)}{Z(t_{seed})}$$

where *t_seed_* was approximately 18 h for CAL-33 and FaDu, and it was approximately 24 h for PE/CA-PJ15, Detroit 562, and hASCs. Normalized technical replicates were subsequently averaged, and data are presented as mean ± SD. One of the four Extract 16-treated CAL-33 wells (B10) was excluded from the ECIS analyses of this plate (see Section 3.3 and Section 3.4.3, Supplementary Figure S3, and Table 4 and Table 5) because of an electrode defect (the electrode appeared partially detached). Dynamic response phenotypes were assigned descriptively based on the treatment-associated impedance trajectory and the presence or absence of recovery after medium replacement. The labels describe impedance behavior and were not interpreted as direct evidence of a specific cytostatic or cytotoxic mechanism.

#### 2.4.2. Frequency-Dependent Analysis and Barrier-Resistance Modeling

The primary ECIS analysis was performed at 16 kHz. To obtain additional frequency-dependent information, normalized impedance responses to Extract 16 were evaluated at 4000 and 64,000 Hz in CAL-33 and hASCs. These are the lowest and highest recorded frequencies and, together with 16 kHz, span the recorded frequency range. Frequency-dependent changes were interpreted descriptively and were not assigned to a specific molecular mechanism without supporting model-based or cell-biological evidence. Barrier resistance (*R*_b_) was obtained from the model-based ECIS analysis implemented in the ECIS software using the recorded multifrequency data. *R*_b_ represents the barrier-associated contribution of cell–cell contacts to the measured impedance [12,13]. Because spectra were recorded only between 4 and 64 kHz, the low-frequency range, in which *R*_b_ contributes most to the impedance [12,13], was not covered; *R*_b_ (Ω·cm^2^), α, and *C*_m_ are therefore semi-quantitative, and *R*_b_ was interpreted as a relative readout within each experiment, comparing Extract 16-treated and DMSO-treated wells on the same array. For each plate, the model was fitted against a reference spectrum from the same plate. Because Extract 16- and DMSO-treated wells were fitted against the same reference, systematic effects of the reference apply equally to both groups; differences between individual electrodes are not corrected by this procedure. Absolute model-derived *R*_b_ values were exported and plotted without normalization. The model was fitted to each time point separately, over the whole recording. Because the model describes a closed cell layer with cell–cell contacts, *R*_b_, α, and *C*_m_ were shown only where the model converged. *R*_b_ values at the lower bound of the software were treated as not modelled, and α and *C*_m_ were not returned at all time points, so the number of modelled wells can differ between parameters. Runs of fewer than 50 consecutive measurements (about 9 h) were regarded as isolated fits and not shown, whereas runs that continued until the end of the recording were shown from 10 consecutive measurements (about 2 h) onward. Runs in which *R*_b_ never exceeded 0.1 Ω·cm^2^ were regarded as fits at the lower bound and not shown; this applied only to Detroit 562 wells. A late onset of convergence in Extract 16-treated wells was kept and reported, because it indicates that the impedance reached the level at which the model resolves *R*_b_ only late in the recovery phase. Mean ± SD was calculated only for time points at which all wells of a condition had been modelled, and end values are given as the mean of the last hour of recording. α and *C*_m_ are shown in Supplementary Figure S10. In Detroit 562, the cell layer raised the impedance only slightly above the cell-free value during the recording (DMSO, about 1.5-fold at 16 kHz and 1.4-fold at 4 kHz at the end of recording, relative to the same well before seeding, compared with 2.2–2.5-fold at 16 kHz in the other cell lines). Within the 4–64 kHz range, *R*_b_ therefore remained at or below 0.1 Ω·cm^2^ in all Detroit 562 wells except one DMSO well at the end of recording, and *R*_b_ was not compared between Extract 16- and DMSO-treated Detroit 562 cells. Extract 16-treated cells were compared with their corresponding DMSO controls within each cellular model. Because baseline *R*_b_ values differed between the HNSCC cell lines, absolute values and temporal trajectories were interpreted within the respective cell line and its DMSO control. hASCs were not included in the *R*_b_ analysis because the model assumes a closed epithelial cell layer with cell–cell contacts, which mesenchymal stem cells do not form. The impedance data of hASCs (Table 4 and Table 5, Supplementary Figures S2 and S3) are not affected.

#### 2.4.3. Quantitative ECIS Metrics

For the quantitative comparison of Extract 16 at 16 kHz, two complementary metrics were calculated from the mean normalized impedance curves.

Treatment-associated difference-in-differences.

$${\Delta \Delta Z}_{treat}=[{Z¯}_{norm,E16}(t_{3})-{Z¯}_{norm,E16}(t_{2})]-[{Z¯}_{norm,DMSO}(t_{3})-{Z¯}_{norm,DMSO}(t_{2})]$$

where *t*_2_ and *t*_3_ denote the cell line-specific start and end of the treatment interval (first measurement after addition of the extract and last measurement before replacement with extract-free medium). Because ΔΔ*Z_treat_* compares only the changes within the treatment interval, differences in normalized impedance that were already present at treatment start do not enter the metric directly.

End-point separation.

$$Z_{end-point\;separation}={Z¯}_{norm,E16}(t_{final})-{Z¯}_{norm,DMSO}(t_{final})$$

Here, ${Z¯}_{norm}$ is the mean of the per-well normalized impedance of all technical replicate wells of a condition, so both metrics are dimensionless. *t_final_* denotes the last measurement of the recording. Negative values indicate lower normalized impedance under Extract 16 relative to the DMSO control. The metrics describe relative impedance behavior and should not be interpreted as direct measures of cell death or proliferation.

Recovery parameters. Because the treatment-containing medium was replaced with extract-free medium at the end of the treatment interval (Section 2.4), the recovery phase was defined as the period from this medium exchange to the end of recording (CAL-33 and FaDu, 65.5–112.0 h; PE/CA-PJ15, 74.3–95.2 h; Detroit 562, 72.2–95.9 h; hASCs, 73.0–97.5 h). Recovery parameters were calculated for each well from the normalized impedance at 16 kHz, for Extract 16 in all cellular models and for the CAL-33 profiles shown in Section 3.3, and are given as mean ± SD of the technical replicate wells (Table 5). The first 2 h after each medium exchange were excluded to avoid handling artifacts. The nadir was defined as the minimum of the normalized impedance (moving average over five measurements, approximately 1 h) after treatment start, and a treatment-associated decline was assumed when the nadir was more than 0.05 below the pre-treatment value (mean of the last three measurements before treatment). The recovery slope was obtained by linear regression over the recovery phase, and the recovery half-time was defined as the time from the nadir until half of the decline had been regained. The residual impedance at the end of recording (mean of the final hour) was expressed relative to the DMSO control after subtraction of the normalized starting level, (*Z*_end_ − 1)/(*Z*_DMSO,end_ − 1) × 100%. Responses were considered persistent when the recovery slope remained close to zero, and the recovery half-time was not reached.

### 2.5. Concentration–Response Experiments with Extract 16 and Inavolisib

To characterize the concentration-dependent metabolic responses to Extract 16, CAL-33, FaDu, PE/CA-PJ15, Detroit 562, and hASCs were seeded in 96-well plates and treated for 24 h with Extract 16 at final concentrations of 3.125, 6.25, 12.5, 25, 50, 100, or 200 µg/mL. Extract 16 working solutions were prepared from a 50 mg/mL DMSO stock solution. The final DMSO concentration was maintained at 0.1% (*v*/*v*) in all Extract 16 treatment and vehicle-control wells. In parallel, concentration-dependent responses to the selective PI3Kα inhibitor Inavolisib (Merck KGaA, Darmstadt, Germany) were assessed using final concentrations of 1, 3, 10, 30, 100, 300, or 1000 nM. The corresponding DMSO-treated cells served as vehicle controls. Cell-free wells containing the extract (blanks) were included as a control to check whether the extract shifted the absorbance; they were not subtracted. After 24 h of treatment, metabolic activity was determined using the CellTiter 96^®^ AQueous One Solution MTS assay as described above. Each concentration was analyzed in four to six technical replicate wells (*n* = 4–6). MTS signals were normalized to the mean DMSO-control signal, which was set to 100%. Concentration–response data were plotted on logarithmic concentration axes and are presented as mean ± SD. Because several treatment profiles were non-monotonic or did not reliably cross the 50% metabolic-activity threshold within the tested concentration range, no forced or extrapolated IC_50_ values were used for the primary interpretation.

Combination experiments were performed in CAL-33 and Detroit 562 cells, which showed the most informative responses to both Extract 16 and Inavolisib in the preceding concentration–response experiments. For CAL-33, cells were treated for 24 h with DMSO, Inavolisib at 1000 nM, Extract 16 at 50 µg/mL, or the combination of 1000 nM Inavolisib and 50 µg/mL Extract 16. The main CAL-33 combination experiment was performed at approximately 70% confluence and included six technical replicate wells per condition (*n* = 6). An additional exploratory experiment was performed at approximately 90–100% confluence to assess the potential influence of cell density on the measured metabolic response. For Detroit 562, cells were treated for 24 h with DMSO, Inavolisib at 1000 nM, Extract 16 at 12.5 µg/mL, or a combination of 300 nM Inavolisib and 12.5 µg/mL Extract 16. Each condition was analyzed using seven technical replicate wells (*n* = 7). After treatment, metabolic activity was measured using the MTS assay. CAL-33 values (Section 3.4.4 and Supplementary Figure S7) were normalized to the mean DMSO-control signal of the same plate without background subtraction; for Detroit 562, the mean signal of the cell-free wells was subtracted before normalization to the mean DMSO-control signal. Individual technical replicate values and boxplots are shown in the Results (Section 3.4.4). The combination experiments were designed as exploratory pharmacological comparisons and were not configured as full concentration matrices for formal Bliss, Loewe, ZIP, or HSA synergy analysis. Consequently, the observed combination responses were interpreted descriptively and were not classified as synergistic, additive, or antagonistic.

### 2.6. Caspase-Glo 3/7 Assay

Caspase 3/7 activity was assessed in preliminary, orienting experiments in CAL-33 and Detroit 562 cells using the Caspase-Glo^®^ 3/7 Assay System (Promega, Madison, WI, USA). Cells were seeded in white, opaque-walled 96-well plates and allowed to attach for 24 h. Cells were subsequently treated with DMSO, Extract 16, Inavolisib, or the corresponding combination using the cell line-specific concentrations of the combination experiments (CAL-33: 50 µg/mL Extract 16, 1000 nM Inavolisib, or 1000 nM Inavolisib plus 50 µg/mL Extract 16; Detroit 562: 12.5 µg/mL Extract 16, 1000 nM Inavolisib, or 300 nM Inavolisib plus 12.5 µg/mL Extract 16). Caspase-Glo 3/7 reagent was prepared and added according to the manufacturer’s instructions. Plates were incubated for 1 h at room temperature while protected from light. Luminescence was measured using a Luminescence 96 microplate reader (Byonoy GmbH, Hamburg, Germany). Cell-free wells containing medium and reagent were included for background correction, and background-corrected luminescence was compared with the corresponding DMSO control. These experiments were intended as a second method for detecting caspase 3/7 activation, because cleaved caspase 3 was not detected by immunoblotting; the results were not evaluable. The assay was used to assess executioner-caspase activity and was not interpreted as a direct measure of cell number or metabolic activity.

### 2.7. Immunofluorescence and Confocal Microscopy

CAL-33 and Detroit 562 cells were seeded on glass coverslips and cultured until the desired confluence was reached. Cells were treated with DMSO, Extract 16, Inavolisib, or the respective combination treatment for 4 or 24 h. The cell line-specific treatment concentrations were 50 µg/mL Extract 16 for CAL-33 and 6.25 µg/mL Extract 16 for Detroit 562; Inavolisib was used at 1000 nM, and the combination treatment consisted of 1000 nM Inavolisib plus the respective Extract 16 concentration. Following treatment, cells were washed three times with PBS and fixed with 4% paraformaldehyde. (Thermo Fisher Scientific, Darmstadt, Germany). Cells were permeabilized with 0.1% Triton X-100 (Thermo Fisher Scientific, Darmstadt, Germany) and blocked for 1 h at room temperature in PBS containing 10% normal goat serum (PAN-Biotech, Aidenbach, Germany) and 0.4% bovine serum albumin (BSA) (Thermo Fisher Scientific, Darmstadt, Germany) F-actin was stained with Phalloidin 488 (sc-363791; Santa Cruz Biotechnology, Dallas, TX, USA) diluted 1:200 in PBS containing 0.4% BSA for 1 h at room temperature. After washing, samples were incubated overnight at 4 °C, protected from light, with primary antibodies diluted 1:200 in PBS containing 0.4% BSA. The following primary antibodies were used: cleaved caspase 3 (catalogue no. 9579; Cell Signaling Technology, Danvers, MA, USA), cleaved PARP (catalogue no. 9544; Cell Signaling Technology), β-catenin (catalogue no. 8480; Cell Signaling Technology), E-cadherin (catalogue no. 3195; Cell Signaling Technology), and ZO-1 (catalogue no. 13663; Cell Signaling Technology). Rabbit primary antibodies were detected using goat anti-rabbit Alexa Fluor 594 secondary antibody (A-11012; Invitrogen, Carlsbad, CA, USA) diluted 1:600 in PBS containing 0.4% BSA for 2 h at room temperature. Samples were mounted using ROTI^®^Mount FluorCare DAPI (HP20.1; Carl Roth, Karlsruhe, Germany) and allowed to cure overnight at 4 °C. Fluorescence images were acquired using a confocal laser-scanning microscope (LSM 900; Carl Zeiss Microscopy GmbH, Jena, Germany) equipped with a 63× oil-immersion objective. Acquisition parameters, including laser power, detector gain, pinhole settings, and image-processing parameters, were kept constant within each marker series. Images were processed using ZEISS ZEN software version 3.13 (Carl Zeiss Microscopy GmbH, Jena, Germany). For most marker series, Phalloidin 488 is displayed in green, the rabbit-primary-antibody signal detected with Alexa Fluor 594 in red, and nuclei are displayed in blue. For presentation of the E-cadherin series, channels were pseudocolored such that E-cadherin is displayed in green and F-actin in red. Because treatment produced marked differences in cell density, morphology, and the number of remaining cells, images were interpreted qualitatively. No quantitative image analysis, such as measurement of fluorescence intensity, colocalization, or cell numbers, was performed; the immunofluorescence data are therefore descriptive. Representative images from two independent biological experiments are shown.

### 2.8. Protein Extraction and Western Blotting

CAL-33 and Detroit 562 cells were seeded in 6-well plates and treated with DMSO, Extract 16, Inavolisib, or the respective combination treatment for 24 h. The cell line-specific concentrations were 50 µg/mL Extract 16, 1000 nM Inavolisib, or 1000 nM Inavolisib plus 50 µg/mL Extract 16 for CAL-33 and 6.25 µg/mL Extract 16, 1000 nM Inavolisib, or 1000 nM Inavolisib plus 6.25 µg/mL Extract 16 for Detroit 562. Following treatment, cells were washed with ice-cold PBS, harvested, and resuspended in 200 µL Bio-Plex™ Cell Lysis Buffer (Bio-Rad Laboratories GmbH, Feldkirchen, Germany). Lysates were incubated on ice for at least 30 min with regular mixing, subjected to eight sonication cycles, and centrifuged at 15,000 rpm for 15 min at 4 °C. The supernatant was transferred to a fresh tube, supplemented with 3 µL magnesium chloride and 3 µL DNase (Thermo Fisher Scientific, Darmstadt, Germany), incubated on ice for 10 min, and centrifuged again at 15,000 rpm for 3 min at 4 °C. Protein concentrations were determined using the Qubit™ Protein BR Assay (Invitrogen, Carlsbad, CA, USA). Lysates were diluted in Laemmli sample buffer (Bio-Rad Laboratories GmbH, Feldkirchen, Germany) to 100 µg protein per 100 µL, denatured for 5 min at 95 °C, and briefly centrifuged. All target proteins, including phosphorylated and total AKT and mTOR, were analyzed in this same set of lysates. Western blotting was performed using the Mini-PROTEAN^®^ Tetra Vertical Electrophoresis Cell and 4–20% Mini-PROTEAN^®^ TGX Stain-Free™ Precast Gels (10-well, 30 µL; cat. no. 4568093; Bio-Rad Laboratories GmbH, Feldkirchen, Germany) for all target proteins. Twenty micrograms of protein and 6 µL Precision Plus Protein™ WesternC™ Blotting Standards (Bio-Rad Laboratories GmbH, Feldkirchen, Germany) were loaded per lane. Electrophoresis was conducted at 60 V for 15 min, followed by approximately 60 min at 160 V. Stain-free gel images were acquired using the ChemiDoc™ XRS+ Imaging System (Bio-Rad Laboratories GmbH, Feldkirchen, Germany) after 300 s UV activation and automatic exposure and served as the lane-wise total-protein loading reference [23]. Proteins were transferred to PVDF membranes (Bio-Rad Laboratories GmbH, Feldkirchen, Germany) in a wet-tank system (Bio-Rad Laboratories GmbH, Feldkirchen, Germany) at 350 mA for 45–60 min with active cooling. Membranes were blocked for 1 h at room temperature in 5% skim milk powder (non-fat dry milk) (Thermo Fisher Scientific) in TBST (ChemCruz, Santa Cruz Biotechnology) and incubated overnight at 4 °C with primary antibodies diluted 1:1000. The analyzed proteins included total PARP (9542; Cell Signaling Technology), cleaved PARP (9544; Cell Signaling Technology), phosphorylated AKT (Ser473; 4060; Cell Signaling Technology), total AKT (9272; Cell Signaling Technology), phosphorylated mTOR (Ser2448; 2971; Cell Signaling Technology), total mTOR (2972; Cell Signaling Technology), total caspase 3 (sc-56052; Santa Cruz Biotechnology), cleaved caspase 3 (9579; Cell Signaling Technology), and β-actin (sc-69879; Santa Cruz Biotechnology). The target proteins were detected on separate gels and membranes; total AKT and total mTOR were analyzed in the same lysates as p-AKT and p-mTOR, but on separate gels and membranes. After four washes with TBST, membranes were incubated for 1 h at room temperature with the appropriate horseradish peroxidase-conjugated secondary antibody diluted 1:5000. Rabbit primary antibodies were detected using goat anti-rabbit IgG-HRP (7074P2; Cell Signaling Technology); the anti-mouse secondary antibody used for β-actin was 7076 from Cell Signaling Technology. Following additional washes, 500 µL of a 1:1 luminol/peroxide chemiluminescent substrate solution SuperSignal™ West Pico PLUS Chemiluminescent Substrate; Thermo Fisher Scientific) was applied, and signals were recorded using the ChemiDoc™ XRS+ Imaging System in Chemi High Sensitivity mode with signal accumulation. Immunoblots are shown as representative images; complete uncropped immunoblots and corresponding stain-free total-protein images are provided in Supplementary Figure S6.

### 2.9. Statistical Analysis

Data visualization and statistical analysis were performed using GraphPad Prism version 8.0.1 (GraphPad Software, San Diego, CA, USA) and Python 3.13 (Python Software Foundation, Wilmington, DE, USA). Data are presented as mean ± standard deviation unless stated otherwise. Biological and technical replicates were distinguished explicitly throughout the manuscript. Technical replicate wells were not considered independent biological observations. The primary MTS screening data were analyzed descriptively and presented as mean ± SD across independent biological experiments. No inferential statistical testing was performed for the complete multi-extract screening dataset. Concentration-response curves for Extract 16 and Inavolisib were plotted on logarithmic concentration axes. IC_50_ values were not forced or extrapolated when the measured response did not reliably cross the 50% metabolic-activity threshold within the investigated concentration range. For the exploratory CAL-33 and Detroit 562 combination experiments, treatment groups were compared using one-way analysis of variance followed by Tukey’s multiple-comparisons test. Because these comparisons were based on technical replicate wells from representative experiments rather than independent biological replicates, the resulting *p*-values were interpreted descriptively. Significance levels were indicated as ns, not significant; *p* < 0.05; *p* < 0.01; and *p* < 0.001. ECIS time courses, frequency-dependent impedance profiles, barrier-resistance trajectories, and derived quantitative metrics were analyzed descriptively. Immunofluorescence experiments and the PARP and phospho-specific Western blots were performed in two independent biological experiments. Total AKT and total mTOR were analyzed in one experiment. No inferential statistical tests were applied to these datasets.

### 2.10. Use of Generative Artificial Intelligence

During manuscript preparation, ChatGPT (GPT-5.6 Pro; OpenAI, San Francisco, CA, USA) was used in the initial phase to support language editing, structural refinement, and code generation for data visualization. In the final revision phase, Grok Bot (version 0.63.0) was used to support the finalization of the manuscript, including text review and formatting. All scientific content, calculations, figures, and interpretations were critically reviewed and validated by the authors, who take full responsibility for the final manuscript.

## 3. Results

### 3.1. MTS-Based Screening Reveals Heterogeneous Extract Sensitivity Across HNSCC Cell Lines

To identify plant extracts with potential tumor-selective activity, all extracts for which suitable assay solutions could be prepared were screened at 50 µg/mL for 24 h using an MTS-based metabolic activity assay in four HNSCC cell lines—CAL-33, FaDu, PE/CA-PJ15, and Detroit 562—and in human adipose-derived stem cells (hASCs) as a non-malignant comparator. As illustrated in Figure 1 for one representative experiment, the response patterns differed markedly among extracts and cellular models. Detroit 562 generally showed pronounced sensitivity to several extracts, whereas CAL-33, FaDu, and PE/CA-PJ15 exhibited more heterogeneous response profiles. Extracts 6, 8, 16, and 19 produced substantial metabolic inhibition in multiple HNSCC cell lines. However, pronounced tumor-cell inhibition was not necessarily associated with preservation of hASC metabolic activity. In particular, the strong effects of Extracts 6, 8, and 19 were accompanied by considerable inhibition in hASCs. By contrast, Extracts 7, 12, 14, 16, and 20 combined measurable metabolic inhibition in one or more HNSCC cell lines with comparatively limited effects on hASCs, indicating varying degrees of tumor selectivity. Negative inhibition values observed for some extract–cell line combinations reflect metabolic activity above the corresponding DMSO-control level and should not be interpreted as negative cytotoxicity. Detailed extract- and cell line-specific MTS results, including mean ± SD values, are provided in Supplementary Figure S1. Overall, the primary MTS screen revealed substantial heterogeneity in extract sensitivity but, as a static endpoint measurement, could not resolve the temporal dynamics, persistence, or reversibility of the observed responses.

### 3.2. Direct Visualization of Tumor Selectivity Identifies Prioritized Extracts

**Figure 2:**
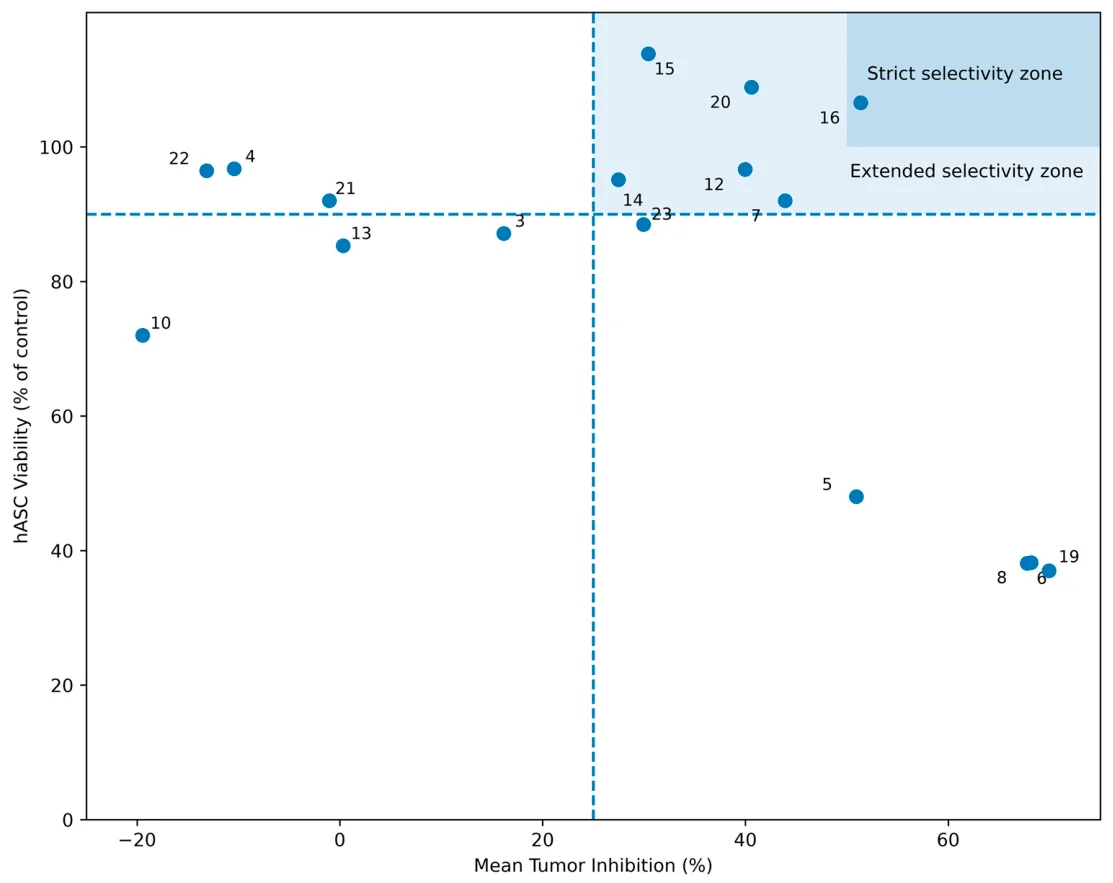
Tumor-selectivity analysis based on MTS screening. Scatter plot showing mean tumor inhibition, calculated as the average inhibition across CAL-33, FaDu, PE/CA-PJ15, and Detroit 562 cells, in relation to the metabolic activity of human adipose-derived stem cells (hASCs) after treatment with the indicated plant extracts at 50 µg/mL for 24 h. Tumor inhibition and hASC metabolic activity were normalized to the respective DMSO controls. Each point represents one extract; the corresponding extract ID is indicated adjacent to the marker. The extended selectivity zone comprises extracts that showed at least 25% mean tumor inhibition while maintaining at least 90% hASC metabolic activity. The strict selectivity zone comprises extracts that showed at least 50% mean tumor inhibition while maintaining hASC metabolic activity at or above 100% of the DMSO control. Dashed blue lines mark the thresholds of 25% mean tumor inhibition and 90% hASC metabolic activity; the light blue area indicates the extended and the darker blue area the strict selectivity zone. Extract 16 was the only extract in the strict selectivity zone. Extracts outside these zones either showed limited mean tumor inhibition or reduced hASC metabolic activity.

To compare antitumor activity with effects on the non-malignant comparator, mean tumor inhibition—calculated as the average inhibition across CAL-33, FaDu, PE/CA-PJ15, and Detroit 562—was plotted against hASC metabolic activity (Figure 2). This representation distinguished extracts with broad, non-selective inhibitory activity from those combining tumor-cell inhibition with preservation of hASC metabolic activity. Two predefined selectivity regions were applied. The extended selectivity zone comprised extracts that produced at least 25% mean tumor inhibition while maintaining hASC metabolic activity at or above 90% of the DMSO control. The strict selectivity zone required at least 50% mean tumor inhibition together with hASC metabolic activity at or above 100% of the DMSO control. Extracts 7, 12, 14, 16, and 20 were located within the extended selectivity zone. Among these candidates, Extract 16 met the criteria for the strict selectivity zone, indicating the most favorable balance between mean HNSCC inhibition and preservation of hASC metabolic activity under the investigated conditions. Because MTS measurements do not distinguish between persistent impedance suppression, heterogeneous responses, or subsequent recovery, selected extracts were further evaluated using real-time ECIS monitoring.

### 3.3. Real-Time Impedance Profiling Reveals Distinct Dynamic Response Phenotypes

**Figure 3:**
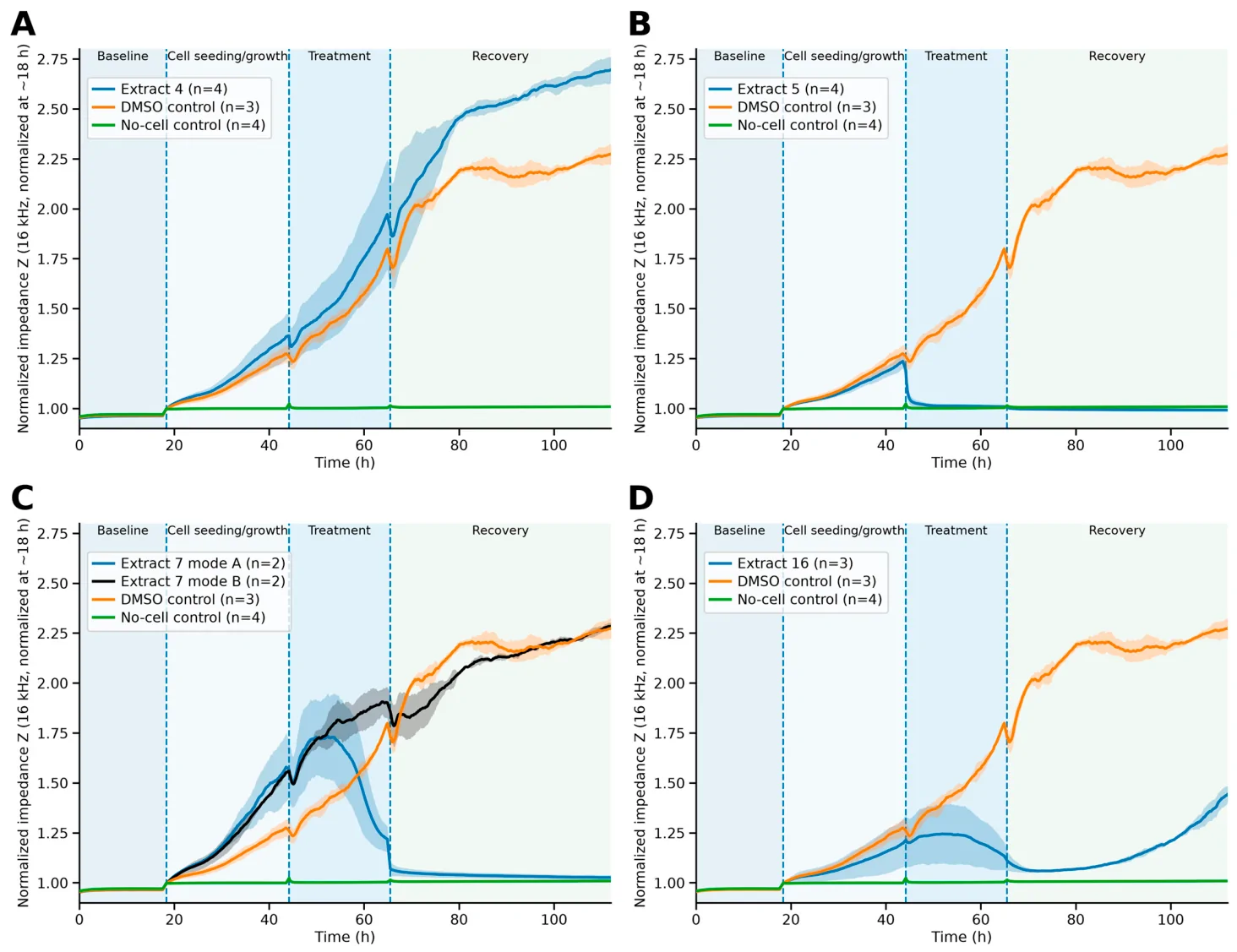
Real-time impedance profiling reveals distinct dynamic response phenotypes in CAL-33 cells. Normalized impedance (*Z* at 16 kHz) was continuously monitored in CAL-33 cells following treatment with selected plant extracts. Impedance values were normalized to the measurement obtained at approximately 18 h. Shaded areas represent mean ± SD of technical replicates. Vertical dashed lines indicate the transitions between baseline, cell seeding/growth, treatment, and recovery phases. (**A**) No growth-inhibitory effect following treatment with Extract 4. Impedance continued to increase after treatment and did not show suppression relative to the DMSO control. (**B**) Sustained impedance suppression following treatment with Extract 5. Impedance decreased rapidly after treatment and remained close to the no-cell control throughout the recovery phase. (**C**) Heterogeneous response to Extract 7. Mode A showed pronounced and sustained impedance suppression, whereas mode B maintained increasing impedance and approached the DMSO-control trajectory during recovery. (**D**) Transient impedance suppression with partial recovery following treatment with Extract 16. Impedance remained below the DMSO control after treatment but increased again during the later recovery phase. Extracts 4 and 5 were analyzed using four technical replicates each (*n* = 4); Extract 7 modes A and B were each analyzed using two technical replicates (*n* = 2); and Extract 16 was analyzed using three technical replicates (*n* = 3). DMSO and no-cell controls comprised three (*n* = 3) and four (*n* = 4) technical replicates, respectively. DMSO controls are shown in orange and no-cell controls in green. Extract-treated cells are shown in blue, except for Extract 7 mode B, which is shown in black.

To complement the endpoint-based metabolic screen, selected extracts were analyzed in CAL-33 cells using ECIS at 16 kHz. Real-time impedance monitoring revealed four distinct dynamic response profiles that could not be differentiated by the MTS endpoint assay alone (Figure 3). Normalized impedance differed between wells already before treatment (1.13–1.67 at treatment start), which we attribute to small differences between individual electrodes, for example, in the efficiency of electrode stabilization or in deposits on the electrode surface. Extract 4 did not produce a growth-inhibitory impedance phenotype. Following treatment, the impedance continued to increase and showed no sustained suppression relative to the DMSO control (Figure 3A). In contrast, Extract 5 induced rapid and persistent impedance suppression, with values remaining close to the no-cell control throughout the recovery phase (Figure 3B). Extract 7 showed heterogeneous responses among technical replicates (Figure 3C). Mode A showed pronounced and sustained impedance suppression, whereas mode B maintained an increasing impedance trajectory and approached the DMSO-control profile during recovery. This divergence demonstrates that identical nominal treatment conditions may produce qualitatively distinct dynamic responses. Both modes persisted when the same wells were analyzed as resistance at 4 kHz and reactance at 64 kHz (Supplementary Figure S9). Extract 16 induced transient impedance suppression followed by partial recovery (Figure 3D). Although impedance remained below the DMSO-control trajectory after treatment, it increased again during the later recovery phase, distinguishing this response from the persistent suppression observed with Extract 5. Together, these findings demonstrate that ECIS provides temporal and phenotypic resolution beyond endpoint metabolic measurements and enables differentiation among sustained, heterogeneous, and partially reversible treatment-associated impedance responses.

### 3.4. Cell Line-Dependent Impedance and Barrier-Resistance Responses to Extract 16

#### 3.4.1. Quantitative Impedance Responses at 16 kHz

To extend the representative CAL-33 response phenotypes to the complete cellular panel, ECIS profiles of selected extracts were compared across CAL-33, FaDu, PE/CA-PJ15, Detroit 562, and hASCs. The complete impedance profiles of Extracts 5, 7, 12, 16, and 20 are provided in Supplementary Figure S2. Extract 16 was selected for quantitative cross-model comparison at 16 kHz. Two complementary metrics were calculated relative to the respective DMSO control: treatment-associated difference-in-differences ΔΔ*Z_treat_*, reflecting the relative impedance change during the cell line-specific treatment interval, and end-point separation, reflecting the difference between Extract 16 and DMSO at the final measurement time (Table 4). Negative values indicate lower normalized impedance under Extract 16 than under the corresponding DMSO control. CAL-33 showed the strongest treatment-associated impedance suppression, with a ΔΔ*Z_treat_* of −0.619 and an end-point separation of −0.831. This profile was characterized by pronounced divergence from the DMSO control during treatment and incomplete convergence during the subsequent recovery phase. In Detroit 562, the treatment-phase difference was less pronounced (ΔΔ*Z_treat_* = −0.102), whereas the end-point separation remained clearly negative (−0.421). This pattern indicates that the divergence from the DMSO control became particularly evident during the later observation period and that recovery-associated impedance development remained limited. FaDu and PE/CA-PJ15 also showed lower normalized impedance relative to their respective DMSO controls, although the magnitude and temporal pattern differed from those observed in CAL-33 and Detroit 562. FaDu exhibited a ΔΔ*Z_treat_* of −0.257 and an end-point separation of −0.461, whereas the corresponding values for PE/CA-PJ15 were −0.240 and −0.224, respectively. In contrast, hASCs showed positive values for both metrics (ΔΔ*Z_treat_* = +0.022; end-point separation = +0.158), indicating that Extract 16 did not produce the impedance suppression observed in CAL-33 (incomplete recovery) and in Detroit 562 (no recovery). Together, these quantitative data demonstrate that Extract 16 induces cell line-dependent impedance responses, with the strongest overall divergence from DMSO observed in CAL-33 and, in Detroit 562, a persistent growth arrest that became apparent mainly after washout. Quantitative recovery parameters separated persistent from transient responses (Table 5): Extract 5 and Extract 7 mode A in CAL-33 and Extract 16 in Detroit 562 showed recovery slopes close to zero and residual impedance of no more than 10% of the DMSO level, whereas Extract 16-treated CAL-33 cells regained half of the impedance decline within 17.9 ± 5.8 h after the nadir but remained at 34 ± 4% of the DMSO level, and FaDu and PE/CA-PJ15 resumed the impedance increase after washout at rates similar to DMSO (67 ± 10% and 78 ± 11% of DMSO at the end of recording).

#### 3.4.2. Frequency-Dependent Impedance Profiles Distinguish CAL-33 and hASC Responses

To obtain additional biophysical information beyond the primary 16-kHz analysis, impedance responses to Extract 16 were examined at 4000, 16,000, and 64,000 Hz in CAL-33 and hASCs (Supplementary Figure S3). In CAL-33, Extract 16 produced impedance trajectories below the corresponding DMSO-control curves at all three investigated frequencies. The magnitude and temporal course of this divergence varied across frequencies, while partial late recovery remained detectable. In hASCs, Extract 16 did not produce a comparable sustained separation from the DMSO control, and the overall trajectories remained more similar to the respective control profiles. These results demonstrate that the response to Extract 16 is detectable across multiple measurement frequencies and differs between CAL-33 and hASCs. However, frequency-dependent impedance data alone do not assign the observed effects to a specific cellular structure or molecular mechanism. Model-based barrier-resistance analysis was therefore used to further characterize the cell line-dependent phenotype.

#### 3.4.3. Barrier Resistance Becomes Resolvable Late or Not at All After Extract 16

**Figure 4:**
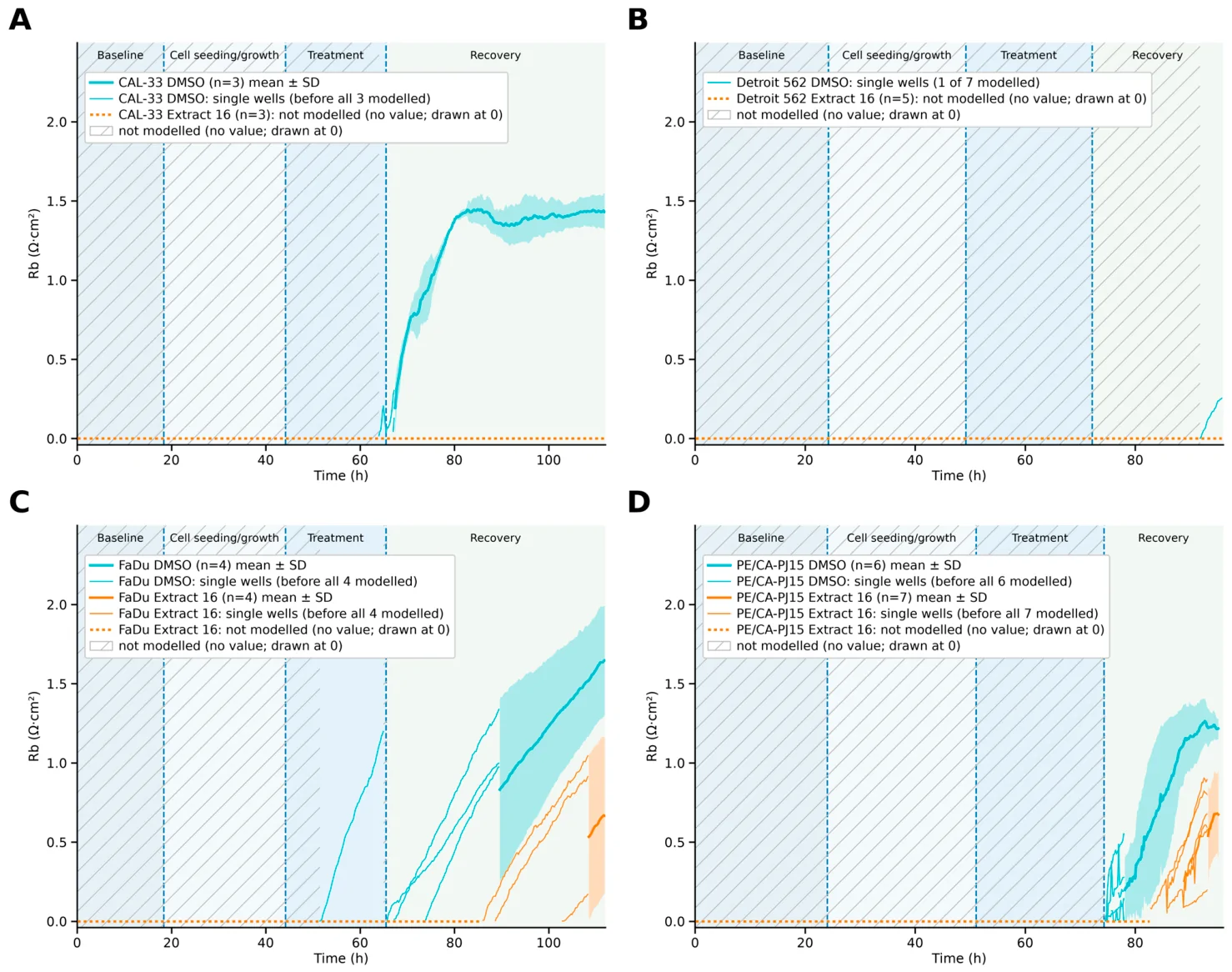
Barrier resistance (*R*_b_) after treatment with Extract 16. *R*_b_ (Ω·cm^2^) was derived from the multifrequency ECIS data (4–64 kHz) by model-based analysis and is shown over the whole recording for Extract 16-treated cells (orange) and the corresponding DMSO controls (turquoise). (**A**) CAL-33, (**B**) Detroit 562, (**C**) FaDu, (**D**) PE/CA-PJ15. Vertical dashed lines mark the plate-specific transitions between baseline, cell seeding/growth, treatment, and recovery (Table 3). *R*_b_ was modelled separately for each well. Thin lines show individual wells; thick lines with shaded areas show the mean ± SD of these wells and are drawn only from the time point at which all wells of a group could be modelled. Because the model describes a closed cell layer with cell–cell contacts, values are shown only where the model converged. Runs of fewer than 50 consecutive measurements (about 9 h) within the recording are not shown; runs that continued until the end of the recording are shown from 10 consecutive measurements (about 2 h) onward. Runs in which *R*_b_ never exceeded 0.1 Ω·cm^2^ were regarded as fits at the lower bound and are not shown (Detroit 562 only). The dotted orange line at 0 marks periods in which no Extract 16 well could be modelled (these are not measured values of zero); grey hatching marks periods in which no DMSO well could be modelled. In CAL-33, the model did not converge in any Extract 16-treated well. In FaDu and PE/CA-PJ15, it converged in the Extract 16-treated wells considerably later than in the DMSO wells (FaDu, 86–109 h vs. 52–90 h; PE/CA-PJ15, 83–93 h vs. 75–78 h), so mean ± SD for Extract 16 covers only the last 3.3 h (FaDu) and 1.9 h (PE/CA-PJ15) of the recording. In Detroit 562, the cell layer raised the impedance only slightly above the cell-free value (DMSO, about 1.5-fold at 16 kHz at the end of recording, compared with 2.2–2.5-fold in the other cell lines). *R*_b_ therefore remained at or below 0.1 Ω·cm^2^ in all wells except one DMSO well (D08), in which it rose during the last 4 h of the recording (0.24 Ω·cm^2^ in the last hour), and *R*_b_ was not compared between Extract 16 and DMSO in this cell line (see Table 4 and Table 5 for the impedance response). Technical replicate wells (Extract 16/DMSO): CAL-33, *n* = 3/3; Detroit 562, *n* = 5/7; FaDu, *n* = 4/4; PE/CA-PJ15, *n* = 7/6.

Model-based analysis of the barrier resistance *R*_b_ complemented the impedance data (Figure 4). The model describes a closed cell layer with cell–cell contacts and resolves *R*_b_ only once the impedance has risen sufficiently above the cell-free level. In the DMSO controls of CAL-33, FaDu, and PE/CA-PJ15, it converged during late treatment or in the recovery phase, and *R*_b_ then increased. In CAL-33, *R*_b_ reached a plateau of about 1.4 Ω·cm^2^ from about 80 h on (1.43 ± 0.11 Ω·cm^2^ at the end of recording); in FaDu and PE/CA-PJ15, it reached 1.64 ± 0.35 and 1.22 ± 0.08 Ω·cm^2^, respectively. In Extract 16-treated CAL-33 cells, the model did not converge in any well until the end of the recording. In FaDu and PE/CA-PJ15, it converged in the Extract 16-treated wells considerably later than in the DMSO wells (FaDu, 86–109 h vs. 52–90 h; PE/CA-PJ15, 83–93 h vs. 75–78 h). *R*_b_ then increased but remained below the DMSO level at the end of recording (FaDu, 0.66 ± 0.50 Ω·cm^2^; PE/CA-PJ15, 0.67 ± 0.26 Ω·cm^2^). In Detroit 562, the cell layer raised the impedance only slightly above the cell-free value (DMSO, about 1.5-fold at 16 kHz at the end of recording, compared with 2.2–2.5-fold in the other cell lines; Extract 16, 1.1-fold). *R*_b_ therefore remained at or below 0.1 Ω·cm^2^ in all wells except one DMSO well (D08), in which it rose during the last 4 h of the recording (0.24 Ω·cm^2^ in the last hour), and *R*_b_ could not be compared between the two groups in this cell line. After Extract 16, the impedance of CAL-33 cells therefore remained below the level at which the model resolves *R*_b_ until the end of the recording, and FaDu and PE/CA-PJ15 reached this level only late. Because the model resolved *R*_b_ only above a similar impedance level in all wells, this pattern reflects the smaller impedance increase; it is consistent with, but does not by itself demonstrate, absent or delayed formation of cell–cell contacts. The parameters α and *C*_m_, obtained from the same fit, are shown in Supplementary Figure S10. Notably, the two cell lines in which impedance remained below the DMSO control after washout, CAL-33 (incomplete recovery) and Detroit 562 (no recovery) (Table 4 and Table 5), are reported to harbor the activating *PIK3CA* p.H1047R variant, whereas FaDu and PE/CA-PJ15 are reported as *PIK3CA* wild-type models (Table 2). This association provided the rationale for subsequent comparison with the selective PI3Kα inhibitor Inavolisib. However, the present data do not establish a causal relationship between *PIK3CA* status and the observed impedance phenotype. Collectively, the quantitative and frequency-dependent impedance analyses, supported by the *R*_b_ data for CAL-33, FaDu, and PE/CA-PJ15, show that Extract 16 produces distinct cell line-dependent impedance phenotypes and identify CAL-33 and Detroit 562 as the most informative models for subsequent pharmacological and cellular validation.

#### 3.4.4. Extract 16 and Inavolisib Exhibit Distinct, Cell Line-Dependent Metabolic Response Profiles

**Figure 5:**
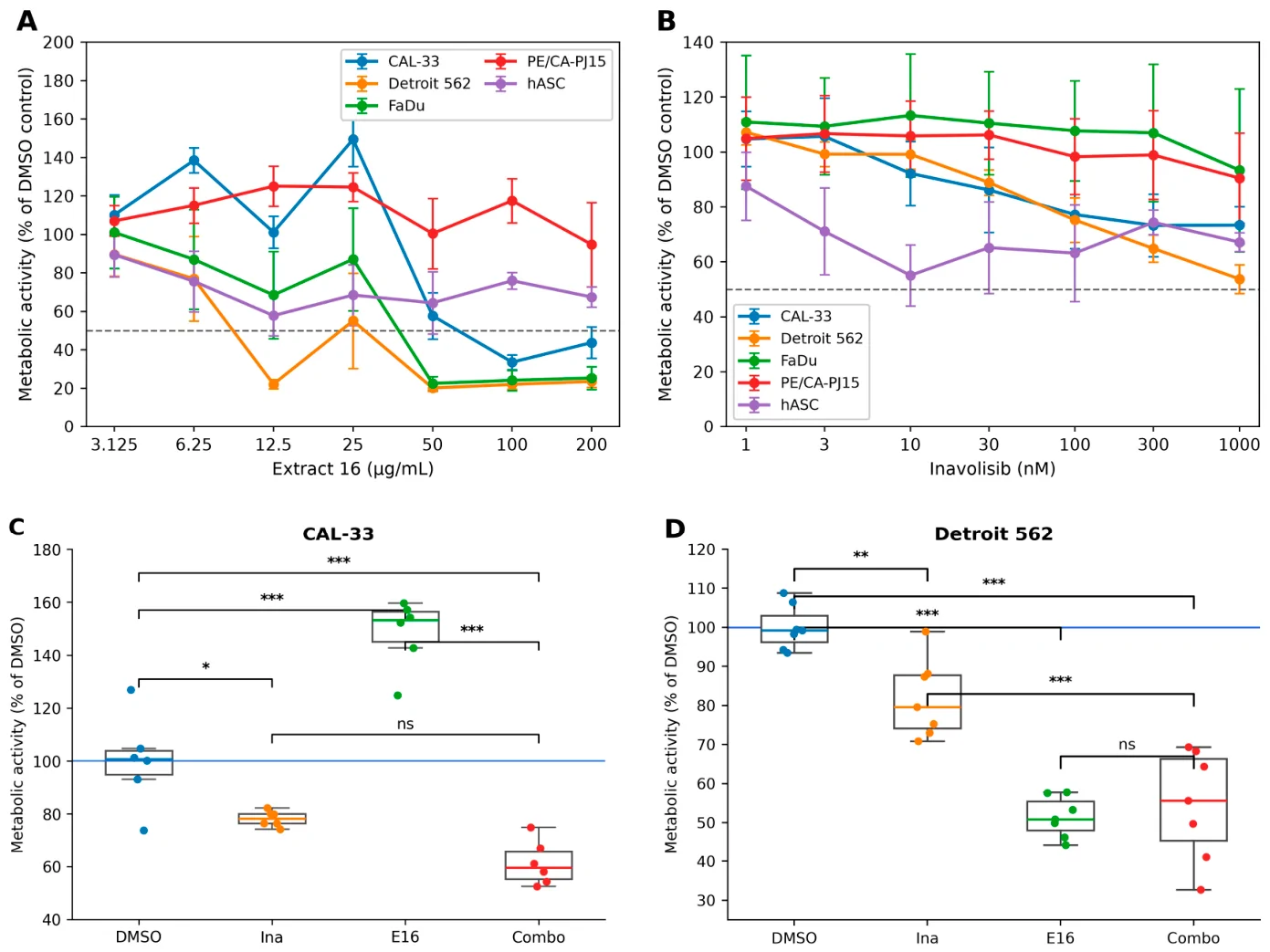
Cell line-dependent metabolic responses to Extract 16 and Inavolisib. (**A**) Concentration-dependent effects of Extract 16 on metabolic activity in CAL-33, Detroit 562, FaDu, and PE/CA-PJ15 HNSCC cells and in human adipose-derived stem cells (hASCs) after 24 h treatment. Extract 16 was tested at concentrations ranging from 3.125 to 200 µg/mL. (**B**) Concentration-dependent effects of Inavolisib on metabolic activity in the same cell lines after 24 h treatment at concentrations ranging from 1 to 1000 nM. In panels (**A**,**B**), metabolic activity was normalized to the respective DMSO control and is presented as the mean ± SD of four to six technical replicate wells per condition (*n* = 4–6). The dashed horizontal line indicates 50% metabolic activity relative to the DMSO control. (**C**) *Metabolic activity* of CAL-33 cells following treatment for 24 h with DMSO, Inavolisib (Ina; 1000 nM), Extract 16 (E16; 50 µg/mL), or their combination (Combo; 1000 nM Inavolisib plus 50 µg/mL Extract 16). Individual values and boxplots from six technical replicates per treatment group are shown (*n* = 6). Panels (**A**,**C**) were obtained in independent experiments performed on different days; the CAL-33 responses to 50 µg/mL Extract 16 in three independent experiments are compared in Supplementary Figure S8. (**D**) *Metabolic activity* of Detroit 562 cells following treatment for 24 h with DMSO, Inavolisib (Ina; 1000 nM), Extract 16 (E16; 12.5 µg/mL), or Combo comprising 300 nM Inavolisib plus 12.5 µg/mL Extract 16. Individual values and boxplots from seven technical replicates per treatment group are shown (*n* = 7). Statistical comparisons in panels (**C**,**D**) were performed using one-way ANOVA followed by Tukey’s multiple-comparisons test. Because these comparisons are based on technical replicates from representative experiments rather than independent biological replicates, the statistical results are presented for descriptive purposes only. Significance levels are indicated as follows: ns, not significant; * *p* < 0.05; ** *p* < 0.01; and *** *p* < 0.001.

To further characterize the pharmacological behavior of the lead candidate, concentration-response experiments were performed for Extract 16 and compared with the PI3Kα inhibitor Inavolisib across the HNSCC cell line panel and hASCs (Figure 5A,B). Extract 16 produced markedly cell line-dependent and partially non-monotonic changes in metabolic activity (Figure 5A). Detroit 562 showed the most pronounced response, with metabolic activity falling to approximately 20–25% of the DMSO control at several concentrations. CAL-33 also showed a substantial reduction at higher concentrations, reaching approximately 34% of the DMSO-control level at 100 µg/mL. FaDu responded predominantly at concentrations of 50 µg/mL and above, whereas PE/CA-PJ15 remained comparatively unaffected across the investigated concentration range. hASCs displayed a variable response, with partial reduction in metabolic activity but without the pronounced suppression observed in Detroit 562. Because several profiles were non-monotonic, the concentration–response data were interpreted descriptively rather than by forcing or extrapolating IC_50_ estimates. Inavolisib produced a more restricted response profile (Figure 5B). *Metabolic activity* progressively declined in CAL-33 and Detroit 562 at increasing concentrations. Detroit 562 showed the strongest response, reaching approximately 54% of the DMSO-control level at 1000 nM, whereas CAL-33 retained approximately 73% metabolic activity at the highest concentration. FaDu and PE/CA-PJ15 remained largely unaffected within the tested range. The hASC response was variable and non-monotonic and therefore did not support a reliable concentration-dependent interpretation. Thus, the response patterns of Extract 16 and Inavolisib partially overlapped in CAL-33 and Detroit 562 but were not identical across the complete cellular panel. Combination experiments were subsequently performed in CAL-33 and Detroit 562 (Figure 5C,D). In the representative CAL-33 experiment, Extract 16 alone increased the normalized MTS signal above the DMSO-control level, whereas Inavolisib reduced metabolic activity. Combo reduced metabolic activity relative to DMSO and Extract 16 alone but did not produce a further reduction relative to Inavolisib alone. An additional exploratory experiment indicated that the relative CAL-33 response to the individual treatments and Combo varied with the degree of cell confluence, identifying cell density or cell state as a relevant source of experimental variability (Supplementary Figure S7). The concentration–response experiment (Figure 5A) and the combination experiment (Figure 5C) were independent experiments performed on different days. At 50 µg/mL, Extract 16 alone reduced the MTS signal of CAL-33 cells to approximately 60% of the DMSO control in the concentration–response experiment, as it did in the primary screen (Figure 1), but increased it above the DMSO-control level in the combination experiment and in the additional experiment at higher confluence, whereas the reductions by Inavolisib and Combo were consistent (Supplementary Figure S8). In Detroit 562, both Inavolisib and Extract 16 reduced metabolic activity relative to DMSO. Extract 16 alone reduced the normalized MTS signal to approximately 51%, whereas Combo produced a similar value of approximately 54%. Combo therefore reduced metabolic activity relative to DMSO and Inavolisib alone but did not enhance the effect of Extract 16 alone. Together, these experiments demonstrate that Extract 16 and Inavolisib produce overlapping but non-identical metabolic response profiles. The combination effects were cell line dependent and did not provide consistent evidence of an enhanced effect relative to both individual treatments. Because the comparisons in Figure 5C,D were based on technical replicates from representative experiments, the associated statistical results were interpreted descriptively and not as confirmatory evidence of pharmacological synergy.

### 3.5. Extract 16 Is Associated with Junctional and Cytoskeletal Remodeling and PARP Processing

**Figure 6:**
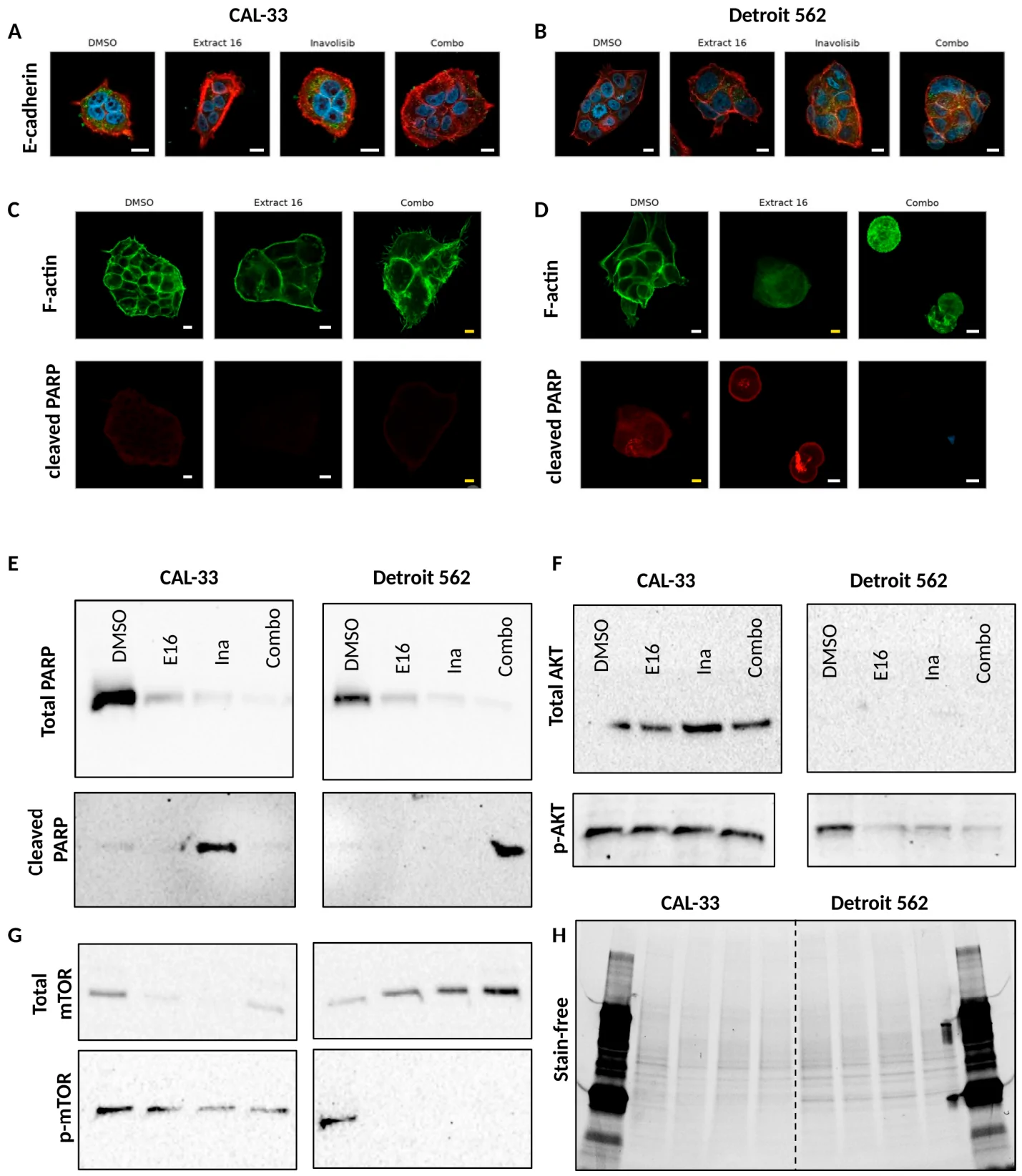
Cell line-dependent junctional, cytoskeletal, and molecular responses to Extract 16 and Inavolisib. (**A**,**B**) Representative confocal images showing E-cadherin distribution in CAL-33 (**A**) and Detroit 562 (**B**) cells following treatment with DMSO, Extract 16, Inavolisib, or the combination treatment (Combo). E-cadherin is shown in green, F-actin in red, and nuclei were counterstained with DAPI. Treatment was associated with altered cellular morphology and redistribution of E-cadherin rather than a uniform loss of E-cadherin immunoreactivity. (**C**,**D**) Representative confocal images showing F-actin organization and cleaved-PARP immunoreactivity in CAL-33 (**C**) and Detroit 562 (**D**) cells following treatment with DMSO, Extract 16, or Combo. Extract 16 and Combo induced cell line-dependent alterations in cell morphology and actin organization. Detroit 562 cells displayed pronounced cell rounding and reorganization of the F-actin cytoskeleton. Cleaved-PARP immunoreactivity was detected in a cell line- and treatment-dependent manner. (**E**–**H**) Representative immunoblots of CAL-33 and Detroit 562 cells treated for 24 h with DMSO, Extract 16 (E16), Inavolisib (Ina), or Combo (CAL-33: 50 µg/mL E16, 1000 nM Ina, Combo 1000 nM Ina plus 50 µg/mL E16; Detroit 562: 6.25 µg/mL E16, 1000 nM Ina, Combo 1000 nM Ina plus 6.25 µg/mL E16). (**E**) Total PARP and cleaved PARP. Treatment was associated with reduced total-PARP band intensity and condition-dependent detection of cleaved PARP. (**F**) Total AKT and p-AKT (Ser473). No specific total-AKT signal was obtained for Detroit 562 under the conditions used; the panel is shown unaltered for completeness. (**G**) Total mTOR and p-mTOR (Ser2448). In Detroit 562, total mTOR was detected under all conditions, whereas p-mTOR was detected only after DMSO. In CAL-33, total mTOR was lower after E16, Ina, and Combo. (**H**) Stain-free total-protein image of the gel used for total mTOR and total AKT, shown as a loading reference. All immunoblots were obtained from the same lysates. Phospho-specific and total proteins were detected on separate gels and membranes, so band intensities cannot be compared directly between panels. Uncropped blots and the stain-free images of all gels are shown in Supplementary Figure S6. Supplementary Figures S4 and S5 complement Figure 6 with further junctional and apoptosis-related markers. Confocal images and the PARP and phospho-specific immunoblots are representative of two independent biological experiments (*n* = 2). Total AKT and total mTOR were detected in one experiment (*n* = 1). Scale bars: white, 10 µm; yellow, 5 µm. The immunoblot data are descriptive and do not establish direct inhibition of the PI3K/AKT/mTOR pathway.

To determine whether the impedance and barrier-resistance phenotypes were accompanied by structural alterations, E-cadherin distribution, F-actin organization, and cleaved-PARP immunoreactivity were examined in CAL-33 and Detroit 562 cells by confocal immunofluorescence microscopy (Figure 6A–D). In DMSO-treated cells, E-cadherin was predominantly localized at cell borders and regions of cell–cell contact. Treatment with Extract 16, Inavolisib, or Combo was associated with changes in cellular morphology and a more heterogeneous spatial distribution of E-cadherin. These alterations were particularly evident in Detroit 562, where treatment produced pronounced changes in cell shape and junctional organization. The images support treatment-associated redistribution of E-cadherin but do not demonstrate a uniform loss of E-cadherin expression. Consistent with this finding, β-catenin and ZO-1 also showed altered spatial organization in Detroit 562 cells (Supplementary Figure S4). F-actin imaging revealed additional cell line-dependent structural responses. CAL-33 cells showed treatment-associated reorganization of the actin cytoskeleton, including changes in cortical actin distribution and cell spreading. In Detroit 562, Extract 16 and Combo produced a more pronounced phenotype characterized by cell rounding, reduced spreading, and disruption of the organized F-actin architecture seen in the DMSO control. Cleaved-PARP immunoreactivity was comparatively limited in CAL-33 but was more apparent in Detroit 562 following Extract 16 treatment. Immunoblotting supported treatment-associated PARP processing, showing reduced total-PARP band intensity and condition-dependent detection of cleaved PARP (Figure 6E). These observations were obtained in two independent biological experiments and were evaluated descriptively, with stain-free total protein as a loading reference. Immunoblotting further revealed treatment-associated changes in p-AKT and p-mTOR, which were interpreted together with total AKT and total mTOR detected in the same lysates (Figure 6F,G). In Detroit 562, total mTOR was detected under all conditions and was, if anything, higher after treatment, whereas p-mTOR (Ser2448) was detected only in DMSO-treated cells. This pattern indicates reduced mTOR phosphorylation after E16, Ina, and Combo. In CAL-33, total mTOR decreased after E16, Ina, and Combo and was nearly undetectable after Ina, so the lower p-mTOR signal in these conditions partly reflects reduced total mTOR and not only reduced phosphorylation. p-AKT (Ser473) remained largely stable in CAL-33 across conditions, while total AKT was, if anything, higher after Ina, so p-AKT relative to total AKT was at most slightly reduced. In Detroit 562, p-AKT was lower after E16, Ina, and Combo than after DMSO. However, no specific total-AKT signal was obtained in Detroit 562 under the conditions used, so p-AKT could not be related to total AKT in this cell line (Figure 6F). Overall, the blots show treatment-associated changes in AKT and mTOR phosphorylation that were more consistent in Detroit 562 than in CAL-33. They do not establish direct inhibition of the PI3K/AKT/mTOR pathway or targeting of *PIK3CA* by Extract 16. Cleaved caspase 3 was not detected by immunoblotting, and the preliminary Caspase-Glo 3/7 experiments did not yield evaluable results. Confocal microscopy likewise showed no consistent treatment-associated increase in cleaved caspase 3 immunoreactivity (Supplementary Figure S5). Collectively, the data indicate that Extract 16 induces a cell line-dependent phenotype involving junctional redistribution, F-actin remodeling, altered barrier-associated behavior, and PARP processing. PARP cleavage was therefore the only apoptosis-associated marker detected (Figure 6C–E), and it depended on the cell line and treatment. Whether caspase-dependent apoptosis is the predominant mode of cell death under the investigated conditions remains open. The structural and biochemical changes were most pronounced in Detroit 562 and were consistent with the persistent impedance suppression in this cell line.

## 4. Discussion

The present study established a multistep functional screening strategy that combines conventional MTS-based endpoint measurements with real-time ECIS monitoring, frequency-dependent impedance analysis, model-derived barrier resistance, and targeted cellular validation. Using a panel of complex plant extracts as a proof-of-concept sample set, the workflow identified substantial extract- and cell line-dependent response heterogeneity and prioritized Extract 16 for extended characterization. The principal methodological finding is that metabolic endpoint measurements and impedance-based live-cell monitoring provide complementary rather than interchangeable information. Whereas MTS screening enabled rapid comparison of a larger extract panel and estimation of relative tumor selectivity, ECIS resolved response kinetics, persistence, heterogeneity, recovery, and barrier-associated behavior that were not apparent from the endpoint assay alone. This distinction is particularly relevant in HNSCC, which comprises a molecularly and phenotypically heterogeneous group of malignancies. Genomic analyses have identified recurrent alterations in *TP53*, *PIK3CA*, cell-cycle regulators, receptor tyrosine kinase signaling, and epithelial differentiation programs, but genomic classification alone does not necessarily predict the functional response of an individual cellular model [2,3,4]. In the present study, the four HNSCC cell lines showed markedly different responses to the same extracts despite standardized experimental conditions. This heterogeneity supports the use of cellular panels rather than a single tumor model when prioritizing complex bioactive samples.

### 4.1. Complementary Information Obtained from MTS and ECIS

The MTS assay was suitable for initial screening because it is technically straightforward, scalable, and sensitive to treatment-associated changes in cellular reducing capacity. However, tetrazolium-based assays quantify metabolic conversion rather than cell number, proliferation, or death directly. *Metabolic activity* may vary independently of cell number and can be influenced by cell density, growth phase, mitochondrial adaptation, and treatment-associated metabolic reprogramming [8]. This limitation was evident in the present concentration-response experiments, in which several cell lines displayed non-monotonic MTS profiles, and CAL-33 showed normalized signals above the DMSO control under some Extract 16 conditions. Such responses should not be interpreted automatically as increased proliferation or treatment resistance. The confluence-dependent CAL-33 experiments further demonstrated that the measured MTS response depended on the cellular state at treatment initiation. In CAL-33, 50 µg/mL Extract 16 lies on the steep part of a biphasic concentration–response curve, where small differences between independent experiments shifted the single 24 h MTS endpoint from a reduction to an increase relative to DMSO (Figure 5A,C and Supplementary Figure S8), and the transient impedance decrease with partial recovery observed by ECIS (Figure 3D) shows that such endpoint values also depend on the time of measurement. Within each experiment, the replicate wells responded uniformly, and in the concentration series, the response changed from 129–163% of DMSO at 25 µg/mL to 44–71% at 50 µg/mL, indicating that the screening concentration lies close to a response threshold. A similar threshold may contribute to the heterogeneous ECIS response of Extract 7 (Section 4.5), although Extract 7 did not diverge in the MTS screen. These observations justify using the term metabolic activity rather than viability throughout the manuscript and explain why forced or extrapolated IC_50_ values were not considered appropriate for several response profiles. ECIS complemented these limitations by enabling continuous, label-free monitoring of adherent cells. Impedance-based biosensors integrate changes in electrode coverage, adhesion, morphology, cell–cell contacts, and membrane-associated electrical properties, thereby providing a dynamic population-level signal without terminating the experiment [10,11,12]. Earlier studies have demonstrated the value of ECIS for evaluating drugs, toxins, purified natural products, and chemically complex extracts, particularly when conventional label-dependent assays may be affected by sample color, redox activity, or direct assay interference [14,24]. Fallarero et al. specifically showed that ECIS could distinguish kinetic cytotoxicity patterns induced by multicomponent natural-product matrices that interfered with conventional assays [14]. The present findings extend this concept to HNSCC models and demonstrate that impedance profiling can refine extract prioritization after an initial metabolic screen. The representative CAL-33 experiments illustrate this added temporal resolution. Extract 4 produced no growth-inhibitory impedance phenotype, Extract 5 caused sustained suppression, Extract 7 generated heterogeneous replicate-dependent responses, and Extract 16 induced transient suppression followed by partial recovery. These profiles would have been reduced to single endpoint values in the MTS assay. In particular, the divergent Extract 7 modes demonstrate that technical or biological heterogeneity can become visible in continuous recordings even when identical nominal treatment conditions are applied. Similarly, the recovery observed after Extract 16 treatment distinguishes a transient perturbation from the persistent response induced by Extract 5. The ability to detect these differences is a major advantage of real-time impedance monitoring in exploratory screening.

### 4.2. Cell Line-Dependent Impedance and Barrier-Resistance Phenotypes

The cross-model ECIS analysis showed that Extract 16 did not produce a uniform response across HNSCC models. CAL-33 displayed the greatest treatment-associated divergence from DMSO at 16 kHz, whereas Detroit 562 showed a more moderate treatment-phase difference but a persistent separation after washout. Both metrics are relative to the DMSO control and therefore also depend on how fast the control grows. In CAL-33, the DMSO control showed the steepest impedance increase of all models during treatment. At the same time, Extract 16 caused a transient decrease with a nadir about 28 h after treatment start, followed by only partial recovery (Table 5). Both contributed to the large divergence. Detroit 562 had grown little when treatment started, and its control increased only slightly during the treatment interval. The separation from DMSO therefore developed mainly after washout, when the control continued to grow, but Extract 16-treated cells did not. This is better described as a persistent growth arrest than as a delayed response. FaDu and PE/CA-PJ15, which are also epithelial, resumed growth after washout at rates close to DMSO. The two lines in which the effect was not fully reversed, CAL-33 and Detroit 562, are both reported to carry *PIK3CA* H1047R. With two lines per group and each line measured on a separate plate, however, genotype cannot be separated from differences in growth kinetics and cell-layer development at the time of treatment. FaDu and PE/CA-PJ15 also exhibited lower normalized impedance than their controls, although their temporal profiles differed. In contrast, hASCs did not show the impedance suppression observed in CAL-33 (incomplete recovery) and in Detroit 562 (no recovery). Thus, Extract 16 was not simply globally toxic under the investigated conditions but generated cell type-dependent dynamic phenotypes. Frequency-dependent measurements provided additional information but were deliberately interpreted conservatively. The contribution of cellular structures to the measured impedance varies with frequency, and multifrequency measurements can separate global impedance from model-derived properties [11,12,13]. Nevertheless, individual frequencies cannot be uniquely assigned to proliferation, membrane integrity, adhesion, or junctional function. The model-derived barrier resistance *R*_b_ provided additional information on cell–cell contacts. *R*_b_ reflects the contribution of cell–cell contacts to current flow and has been used to characterize junctional integrity in adherent cell layers [12,13]. In Extract 16-treated CAL-33 cells, the impedance remained below the level at which the model resolves *R*_b_ until the end of the recording, whereas FaDu and PE/CA-PJ15 reached it considerably later than their controls and then showed lower *R*_b_. Because this level was similar in all wells, the onset of modelled *R*_b_ is not independent of the impedance data, but it is consistent with delayed formation of cell–cell contacts. In Detroit 562, even the DMSO-treated cell layer raised the impedance only slightly above the cell-free value, so *R*_b_ could not be resolved within the 4–64 kHz range. The persistent effect in this line rests on the impedance metrics and the growth arrest after washout described above, not on *R*_b_. Absolute *R*_b_ values were low, and we interpret *R*_b_ as a relative readout within each plate (Section 2.4.2). The immunofluorescence data point in the same direction. They showed treatment-associated redistribution of E-cadherin, β-catenin, and ZO-1 and reorganization of F-actin, particularly in Detroit 562. E-cadherin–catenin complexes are physically and functionally connected to the actin cytoskeleton, and changes in their localization can alter epithelial architecture and cell–cell adhesion without requiring a uniform reduction in total protein abundance [25]. Accordingly, the present images are interpreted as junctional redistribution and cytoskeletal remodeling rather than proof of epithelial–mesenchymal transition or loss of marker expression.

### 4.3. Relationship to PIK3CA Status and Inavolisib Response

The incomplete recovery in CAL-33 and the absent recovery in Detroit 562, together with the absence of resolvable *R*_b_ in Extract 16-treated CAL-33 cells, were of particular interest because both models have been reported to harbor activating *PIK3CA* H1047R variants, whereas FaDu and PE/CA-PJ15 are generally used as *PIK3CA*-wild-type comparators [4]. PI3K-pathway alterations occur frequently in HNSCC and have been proposed as predictive biomarkers for PI3K-pathway-directed treatment [3,4]. Previous work has also shown that endogenous *PIK3CA* H1047R-mutant CAL-33 and Detroit 562 cells can display increased sensitivity to PI3K-pathway inhibition compared with selected wild-type models [4]. These observations provided the rationale for comparing Extract 16 with Inavolisib. Inavolisib is a highly selective ATP-competitive PI3Kα inhibitor that can additionally promote degradation of mutant PI3Kα under appropriate receptor tyrosine kinase signaling conditions [26]. In the present experiments, Inavolisib reduced metabolic activity primarily in CAL-33 and Detroit 562, whereas FaDu and PE/CA-PJ15 remained largely unaffected within the tested range. This partial alignment with the reported *PIK3CA* status supports the biological relevance of the cellular panel. However, Extract 16’s response profile did not fully reproduce that of Inavolisib. Extract 16 also affected FaDu at higher concentrations, induced non-monotonic MTS responses, and produced distinct ECIS and *R*_b_ profiles. Moreover, the combination of Extract 16 and Inavolisib did not consistently enhance the effect of both single treatments: in CAL-33, Combo did not outperform Inavolisib alone, whereas in Detroit 562 it did not exceed the effect of Extract 16 alone. These data argue against interpreting Extract 16 as a conventional PI3Kα inhibitor. The reductions in p-AKT and p-mTOR, particularly in Detroit 562, are compatible with treatment-associated modulation of this pathway but do not establish direct target engagement. In Detroit 562, total mTOR remained detectable under all conditions, whereas p-mTOR was lost after treatment, which points to reduced mTOR phosphorylation. In CAL-33, by contrast, total mTOR itself decreased, and p-AKT was largely unchanged, so the phospho-signals in this cell line cannot be read as reduced phosphorylation alone. Whether p-AKT is reduced relative to total AKT in Detroit 562 remains open, because total AKT was not detectable in this cell line under the conditions used. In addition, Extract 16 is a chemically complex mixture and may influence several signaling and structural pathways simultaneously. The Inavolisib comparison should therefore be interpreted as a pharmacological reference rather than as mechanistic proof.

### 4.4. PARP Cleavage Without Detectable Caspase 3/7 Activation

Extract 16 treatment was associated with cleaved-PARP immunoreactivity and reduced total-PARP band intensity, particularly in Detroit 562. However, caspase 3/7 activation could not be detected: cleaved caspase 3 was not detected by immunoblotting, the preliminary Caspase-Glo 3/7 experiments did not yield evaluable results, and confocal microscopy showed no consistent increase in cleaved caspase 3 immunoreactivity. Apoptosis-associated PARP cleavage was thus detected in a cell line- and treatment-dependent manner, but whether caspase-dependent apoptosis is the predominant mode of cell death under the investigated conditions remains open. PARP cleavage is often used as an apoptosis-associated marker because activated caspases 3 and 7 can generate the characteristic 89-kDa PARP fragment. However, PARP can also be processed by other proteases, including calpains, cathepsins, granzymes, and matrix metalloproteinases, and PARP cleavage alone does not identify the responsible death pathway [27]. Current recommendations for programmed-cell-death analysis emphasize the use of multiple complementary readouts rather than classification from a single marker [28]. The present combination of PARP processing, F-actin remodeling, and altered cell morphology, without detectable caspase 3/7 activation, is therefore best described as a stress- or cell-death-associated phenotype whose precise mechanism remains unresolved. Additional studies addressing membrane integrity, mitochondrial depolarization, alternative caspases, calpain or cathepsin activity, and regulated non-apoptotic death pathways would be required for definitive classification.

### 4.5. Relevance of Complex Extracts and Workflow-Based Screening

Natural products remain an important source of structurally and biologically diverse pharmacophores, but chemically complex extracts pose substantial challenges for screening, reproducibility, dereplication, target identification, and standardization [6]. The seed material used for Extract 16 was re-authenticated as *Ricinodendron heudelotii* (Baill.) Pierre ex Heckel and linked to voucher specimen UBH-R623. However, the chemical composition of the biologically tested dichloromethane batch was not characterized within the present study. A published GC-MS profile is available only for the n-hexane extract of the same seed material (Extract 7) and showed predominantly fatty acids, with oleic acid as the main constituent [29]. Extract 16 should therefore be regarded as a functionally prioritized complex sample rather than a chemically defined therapeutic candidate. This limitation does not invalidate the principal methodological conclusion. A functional phenotypic workflow can be valuable before complete compound identification, provided that sample preparation and batch traceability are documented and biological claims remain proportionate to the available chemical information. The present workflow progressively reduced a larger extract set by integrating tumor/non-malignant metabolic screening, real-time impedance phenotyping, quantitative and frequency-dependent analysis, barrier modeling, pharmacological comparison, and cellular validation. Extract 7, the n-hexane extract of the same seed material, was not pursued further because its ECIS response was heterogeneous between replicate wells (Figure 3C). Possible explanations, such as uneven dispersion of lipophilic constituents in the medium or a concentration close to a threshold at which small differences between wells decide the response, were not tested. This strategy may help identify extracts or fractions that merit subsequent chemical characterization and may also reveal phenotypes missed by endpoint screening alone.

### 4.6. Strengths and Limitations

A major strength of the study is the integration of multiple orthogonal readouts. The MTS assay enabled broad screening, ECIS provided continuous kinetic information, model-based analysis provided supportive, semi-quantitative information on cell–cell contacts, and immunofluorescence and Western blotting provided structural and biochemical context. The use of four HNSCC models and hASCs as a non-malignant comparator further reduced the risk of basing prioritization on a single cell line. Several limitations should nevertheless be considered. First, hASCs are mesenchymal stromal cells and not a tissue-matched epithelial control. Tumor selectivity in this study therefore refers only to the comparison with this non-malignant reference and not to normal oral mucosa; this should be addressed with non-malignant oral epithelial cells in future work. As with all primary cells, the response of hASCs may also depend on the donor. Second, the initial extract panel was tested at a single nominal concentration and time point, and four extracts could not be evaluated because suitable solutions could not be prepared. Third, the complex extract composition may have directly affected metabolic readouts, although cell-free controls and ECIS reduced this concern. No residual-solvent analysis was performed, and no extraction blanks were tested, so contributions of extraction residues cannot be excluded. Fourth, combination experiments were based on technical replicates from representative experiments and were not designed as formal synergy matrices. Fifth, immunofluorescence and the PARP and phospho-specific Western blots were performed in two independent biological experiments, and total AKT and total mTOR in one, permitting descriptive but not robust inferential analysis. Sixth, total AKT and total mTOR were detected on separate gels and membranes from the same lysates. No specific total-AKT signal was obtained in Detroit 562, and total mTOR decreased in treated CAL-33 cells, so phosphorylation relative to total protein could be assessed only for part of the conditions. The total-protein blots should be confirmed in lysates from independent treatments. Seventh, spectra were recorded only between 4 and 64 kHz, so the model-derived *R*_b_, α, and *C*_m_ are semi-quantitative and were interpreted only relative to the DMSO control on the same array. Finally, the biologically tested Extract 16 batch was not chemically characterized, for example, by LC-MS/MS, and the constituents responsible for the observed effects are unknown. These limitations should be addressed in subsequent fractionation, chemical fingerprinting, and validation studies.

## 5. Conclusions

The present study demonstrates that conventional metabolic endpoint screening and real-time impedance monitoring provide complementary information for the functional prioritization of complex plant extracts. MTS enabled rapid comparison of extract activity and relative tumor selectivity, whereas ECIS resolved treatment kinetics, response heterogeneity, persistence, recovery, and barrier-associated behavior. Extract 16 emerged as the most extensively characterized candidate and produced cell line-dependent impedance, junctional, cytoskeletal, and biochemical responses. Impedance remained below the DMSO control after washout in CAL-33 (incomplete recovery) and Detroit 562 (no recovery), and barrier resistance became resolvable only late in FaDu and PE/CA-PJ15 and not at all in CAL-33 after Extract 16, while hASCs did not show comparable sustained impedance suppression. The response profile of Extract 16 only partially overlapped with that of Inavolisib, and combination treatment did not consistently enhance the effects of both single treatments. Apoptosis-associated PARP cleavage was detected in a cell line- and treatment-dependent manner, whereas caspase 3/7 activation could not be detected; whether caspase-dependent apoptosis predominates, and thus the underlying stress- or cell-death-associated mechanism, remains unresolved. Because the same extracts produced transient, persistent, and heterogeneous impedance phenotypes that single MTS endpoints did not resolve, and because the MTS endpoint itself changed direction between experiments, live-cell monitoring should be used more routinely alongside endpoint assays to follow responses during treatment, in screening and in other research applications. Overall, the integrated workflow provides a proof of concept for using label-free ECIS monitoring to refine conventional endpoint screening and to prioritize complex bioactive samples for subsequent chemical fractionation, compound identification, and mechanistic validation.

## Figures and Tables

**Table 1:** Plant extracts included in the cell-based screening. Botanical source, plant part, extraction solvent, physical state, and voucher-specimen information are provided for all extracts included in the biological assays. All extracts were prepared as 50 mg/mL stock solutions in DMSO and diluted 1:1000 in assay medium for the primary screen, yielding 50 µg/mL extract and 0.1% (*v*/*v*) DMSO. Extracts 9, 11, 17, and 18 were excluded because suitable assay solutions could not be prepared owing to insufficient solubility. Extracts 7 and 16 correspond to the n-hexane and dichloromethane seed extracts, respectively, of the re-authenticated *Ricinodendron heudelotii* material (voucher specimen UBH-R623).

Extract ID	Sample Code	Botanical Name	Plant Part	Extraction Solvent	Physical State	Voucher Specimen No.	Stock Solution
3	UL-H-03	*Uapaca lissopyrena*	Fruits	n-Hexane	Oil	UBH-U621	50 mg/mL in DMSO
4	MM-H-04	*Monodora myristica*	Seeds	n-Hexane	Oil	UBH-M350	50 mg/mL in DMSO
5	AS-H-05	*Aframomum sceptrum*	Seeds	n-Hexane	Oil	UBH-A620	50 mg/mL in DMSO
6	XA-H-06	*Xylopia aethiopica*	Seeds	n-Hexane	Oil	UBH-X348	50 mg/mL in DMSO
7	CA-H-07	*Ricinodendron heudelotii*	Seeds	n-Hexane	Oil	UBH-R623	50 mg/mL in DMSO
8	PG-H-08	*Piper guineense*	Seeds	n-Hexane	Oil	UBH-P315	50 mg/mL in DMSO
10	AL-D-01	*Afrostyrax lepidophyllus*	Seeds	Dichloromethane	Solid	UBH-A622	50 mg/mL in DMSO
12	UL-D-03	*Uapaca lissopyrena*	Fruits	Dichloromethane	Oil	UBH-U621	50 mg/mL in DMSO
13	MM-D-04	*Monodora myristica*	Seeds	Dichloromethane	Oil	UBH-M350	50 mg/mL in DMSO
14	AS-D-05	*Aframomum sceptrum*	Seeds	Dichloromethane	Oil	UBH-A620	50 mg/mL in DMSO
15	XA-D-06	*Xylopia aethiopica*	Seeds	Dichloromethane	Solid	UBH-X348	50 mg/mL in DMSO
16	CA-D-07	*Ricinodendron heudelotii*	Seeds	Dichloromethane	Oil	UBH-R623	50 mg/mL in DMSO
19	AM-D-10	*Aframomum melegueta*	Seeds	Dichloromethane	Oil	UBH-A471	50 mg/mL in DMSO
20	TT-D-11	*Tetrapleura tetraptera*	Fruits	Dichloromethane	Solid	UBH-T472	50 mg/mL in DMSO
21	JS-D-12	*Justicia* spp.	Seeds	Dichloromethane	Solid	UBH-1R627	50 mg/mL in DMSO
22	JS-M-01	*Justicia* spp.	Seeds	Methanol	Solid	UBH-1R627	50 mg/mL in DMSO
23	AM-M-02	*Aframomum melegueta*	Seeds	Methanol	Solid	UBH-A471	50 mg/mL in DMSO

**Table 2:** Selected molecular characteristics of the HNSCC cell lines and the non-malignant hASC comparator used in this study. Molecular annotations were derived from the published cell-line databases and literature and were not independently determined in the present study. HPV, human papillomavirus; SCC, squamous cell carcinoma; hASC, human adipose-derived stem cell. Molecular annotations were compiled from Cellosaurus and the published literature [4,15,16,17,18].

Cell Line	HPV Status	*TP53* Status	*PIK3CA* Status	Relevant Molecular Context	Origin
CAL-33	Negative	p.R175H	p.H1047R	Activating *PIK3CA* hotspot mutation	Oral tongue SCC
FaDu	Negative	p.R248L; additional splice-site alteration	Wild type	*PIK3CA*-wild-type comparator	Hypopharyngeal SCC
PE/CA-PJ15	Negative	p.R248Q (c.743G > A)	Wild type	*PIK3CA*-wild-type comparator	Oral tongue SCC
Detroit 562	Negative	p.R175H	p.H1047R	Activating *PIK3CA* hotspot mutation	Pharyngeal SCC; derived from metastatic pleural effusion
hASC	n/a	Not assessed	Not assessed	Non-malignant comparator	Adipose-derived stem cells

**Table 3:** Plate-specific ECIS phase definitions used for the respective cellular models.

Cellular Model	Baseline	Cell Seeding/Growth	Treatment	Recovery
CAL-33	0–18 h	18–44 h	44–65 h	>65 h
FaDu	0–18 h	18–44 h	44–65 h	>65 h
PE/CA-PJ15	0–24 h	24–50 h	50–74 h	>74 h
Detroit 562	0–24 h	24–49 h	49–72 h	>72 h
hASC	0–24 h	24–49 h	49–73 h	>73 h

**Table 4:** Quantitative impedance metrics for Extract 16 at 16 kHz across cellular models. ΔΔ*Z_treat_* represents the treatment-associated difference-in-differences between Extract 16 and the corresponding DMSO control during the cell line-specific treatment interval. End-point separation represents the difference in normalized impedance between Extract 16 and DMSO at the final measurement time. Negative values indicate lower normalized impedance under Extract 16 than under DMSO. Metrics were calculated from the mean normalized impedance curves. Technical replicate numbers for Extract 16/DMSO were CAL-33, *n* = 3/3; FaDu, *n* = 4/4; PE/CA-PJ15, *n* = 7/6; Detroit 562, *n* = 5/7; and hASCs, *n* = 4/4. Reported *PIK3CA* status is provided for molecular context and was not independently determined in the present study.

Cell Line	*PIK3CA* Status	ΔΔ*Z_treat_* (vs. DMSO)	End-Point Separation (Extract–DMSO)
CAL-33	p.H1047R	−0.619	−0.831
FaDu	Wild type	−0.257	−0.461
PE/CA-PJ15	Wild type	−0.240	−0.224
Detroit 562	p.H1047R	−0.102	−0.421
hASC	Not assessed	+0.022	+0.158

**Table 5:** Recovery parameters derived from the normalized impedance at 16 kHz. Nadir, minimum of the normalized impedance after treatment start, with its time after treatment start in parentheses; “no decline”, nadir not more than 0.05 below the pre-treatment value. Nadir times of 67–68 h in CAL-33 correspond to the end of recording (continuous decline without recovery). Recovery slope, linear regression over the recovery phase after medium exchange. Recovery half-time, time from the nadir until half of the decline had been regained; n.r., not reached by the end of recording; –, not applicable. Residual impedance, (*Z*_end_ − 1)/(*Z*_DMSO,end_ − 1) × 100% at the end of recording. Data are presented as mean ± SD of technical replicate wells (*n*).

Cell Line	Treatment	*n* (Wells)	Nadir *Z* (Time After Treatment Start, h)	Recovery Slope (×10^−3^ h^−1^)	Recovery Half-Time (h)	Residual Impedance (% of DMSO)
CAL-33	Extract 4	4	no decline	13.3 ± 3.8	–	133 ± 5
	Extract 5	4	0.99 ± 0.01 (67.8 ± 0.0)	−0.1 ± 0.0	n.r.	−1 ± 1
	Extract 7 mode A	2	1.03 ± 0.01 (66.8 ± 1.5)	−0.5 ± 0.2	n.r.	2 ± 1
	Extract 7 mode B	2	no decline	9.8 ± 2.3	–	101 ± 1
	Extract 16	3	1.06 ± 0.01 (28.5 ± 2.6)	7.8 ± 0.8	17.9 ± 5.8	34 ± 4
	DMSO	3	no decline	5.6 ± 1.7	–	100 ± 4
FaDu	Extract 16	4	no decline	15.1 ± 3.7	–	67 ± 10
	DMSO	4	no decline	17.6 ± 3.3	–	100 ± 7
PE/CA-PJ15	Extract 16	7	no decline	27.6 ± 6.6	–	78 ± 11
	DMSO	6	no decline	23.3 ± 7.6	–	100 ± 1
Detroit 562	Extract 16	5	no decline	1.4 ± 1.0	–	10 ± 7
	DMSO	7	no decline	15.0 ± 2.5	–	100 ± 16
hASC	Extract 16	4	no decline	−1.5 ± 3.5	–	130 ± 22
	DMSO	4	no decline	2.9 ± 3.6	–	100 ± 14

## Data Availability

The data presented in this study are available from the corresponding author upon reasonable request. Cell-biological data are available from N.E.; information and source data relating to plant material and extraction are available from V.O.I.

## Supplementary Materials

The following supporting information can be downloaded at https://www.mdpi.com/article/10.3390/bios16100563/s1. Figure S1. Extract- and cell line-dependent changes in metabolic activity following MTS screening. Figure S2. Real-time ECIS impedance profiles of selected plant extracts across HNSCC cell lines and hASCs. Figure S3. Frequency-dependent impedance responses to Extract 16 in CAL-33 cells and hASCs. Figure S4. Treatment-associated redistribution of β-catenin and ZO-1 in Detroit 562 cells. Figure S5. No treatment-associated increase in cleaved caspase 3 immunoreactivity in CAL-33 and Detroit 562 cells. Figure S6. Original immunoblot and stain-free total-protein images. Figure S7. Exploratory comparison of CAL-33 metabolic responses at different levels of cell confluence. Figure S8. Metabolic response of CAL-33 cells to Extract 16 in three independent MTS experiments. Figure S9. Resistance at 4 kHz and reactance at 64 kHz of CAL-33 cells treated with Extract 7. Figure S10. Model-derived α and membrane capacitance (*C*_m_) of CAL-33, Detroit 562, FaDu, and PE/CA-PJ15 cells treated with Extract 16.
