# Supplementary material for: Multiparametric ECIS Profiling Complements Metabolic Endpoint Screening of Complex Plant Extracts in HNSCC Models
Source: Biosensors (Basel). 2026 Oct 5;16(10):563. doi: 10.3390/bios16100563 (PMC13702393; doi:10.3390/bios16100563)

Supplementary Figure S1. Extract- and cell line-dependent changes in metabolic activity following MTS screening. Changes in metabolic activity after treatment with the indicated plant extracts at 50 µg/mL for 24 h are shown for CAL-33 (A), FaDu (B), PE/CA-PJ15 (C), Detroit 562 (D), and human adipose-derived stem cells (hASC; E). Metabolic activity was measured using the MTS assay and normalized to the corresponding DMSO control. Results are expressed as the percentage-point difference from the DMSO control, which was defined as zero. Negative values indicate reduced metabolic activity relative to DMSO, whereas positive values indicate metabolic activity above the respective control level. Bars represent mean ± SD. CAL-33, FaDu, PE/CA-PJ15, and Detroit 562 were analyzed in three independent biological experiments each (n=3); hASC were analyzed in two independent biological experiments (n=2). Extracts for which suitable assay solutions could not be prepared because of insufficient solubility were not included. normalized to zero. Statistical analysis was performed using the t-test: (*) p < 0.05; (**) p < 0.01; ns = not significant

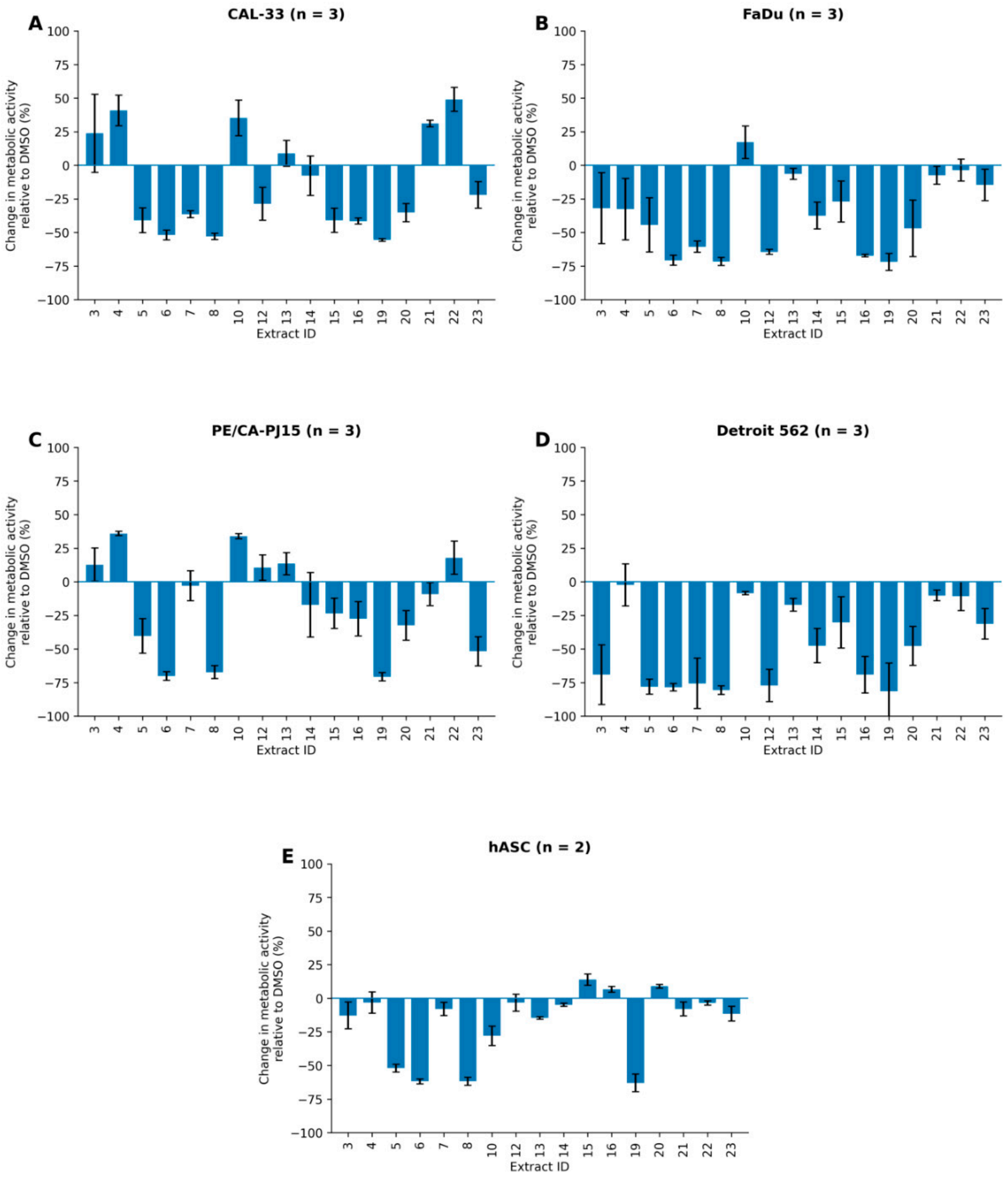

Supplementary Figure S2. Real-time ECIS impedance profiles of selected plant extracts across HNSCC cell lines and hASC. Impedance changes at 16 kHz were continuously monitored in FaDu, CAL-33, PE/CA-PJ15, and Detroit 562 HNSCC cells and in human adipose-derived stem cells (hASC) following treatment with Extracts 5, 7, 12, 16, and 20. Curves represent mean impedance change ± SD of technical replicates. The corresponding DMSO-treated cell control and no-cell control are included in each panel. Vertical dashed lines indicate the plate-specific transitions between baseline, cell seeding/growth, treatment, and recovery phases. Phase boundaries were 18, 44, and 65 h for CAL-33 and FaDu; 24, 50, and 74 h for PE/CA-PJ15; 24, 49, and 72 h for Detroit 562; and 24, 49, and 73 h for hASC. The figure provides a descriptive overview of extract- and cell line-dependent impedance dynamics, including differences in treatment-associated suppression, response heterogeneity, and recovery behavior. Because ECIS kinetics and Y-axis ranges differ between experimental plates, absolute signal magnitudes should be interpreted within the respective cell-line and control context.

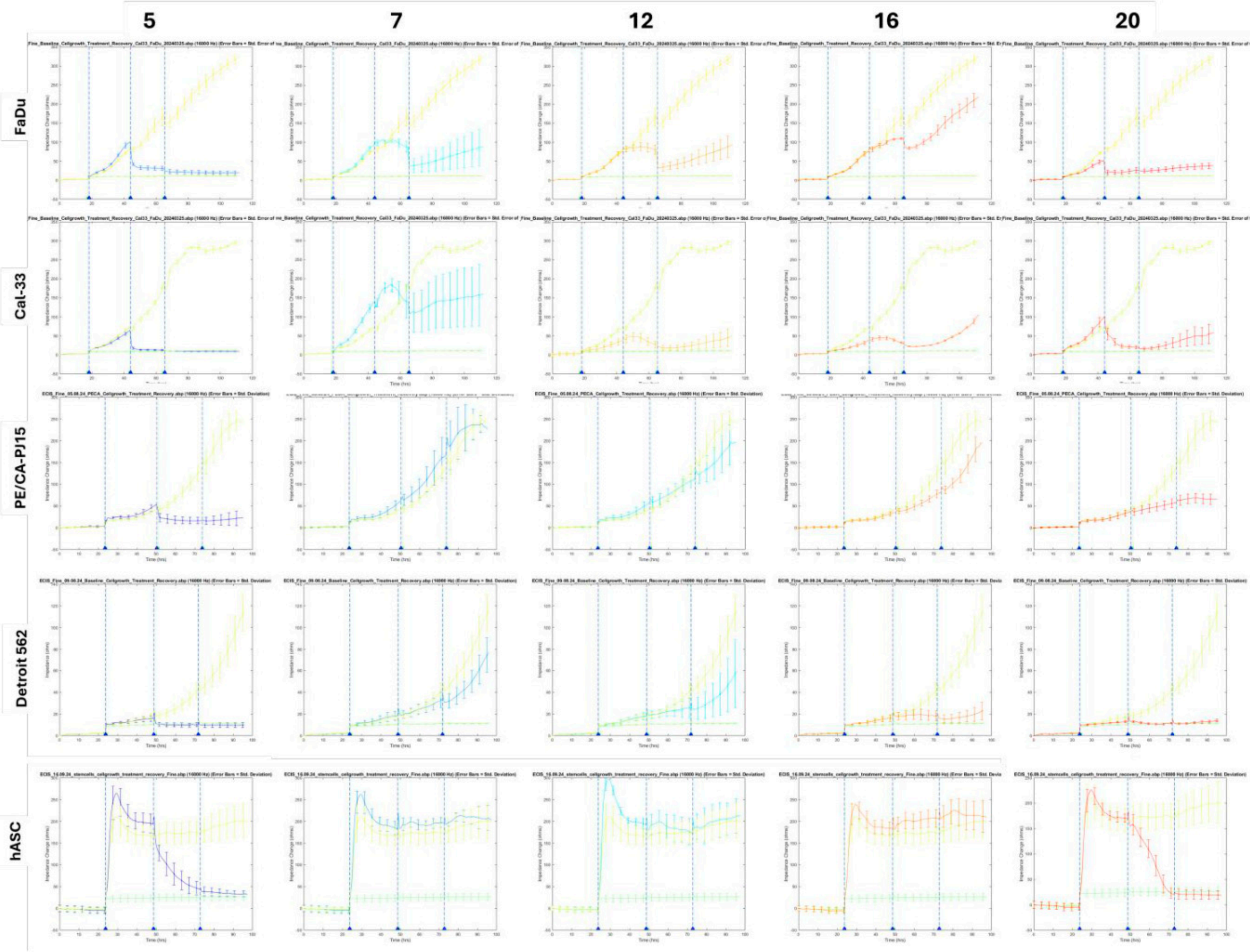

Supplementary Figure S3. Frequency-dependent impedance responses to Extract 16 in CAL-33 cells and hASC. Normalized impedance (Z) was continuously analyzed at 4000, 16000, and 64000 Hz in CAL-33 cells and human adipose-derived stem cells (hASC) following treatment with Extract 16. Impedance values were normalized to the respective cell-seeding time point, corresponding to approximately 18 h for CAL-33 and 24 h for hASC. Curves represent mean ± SD of technical replicates. For CAL-33, Extract 16 was analyzed using three technical replicates (n=3); the DMSO and no-cell controls comprised three (n=3) and four (n=4) technical replicates, respectively. For hASC, Extract 16, DMSO, and no-cell controls were each analyzed using four technical replicates (n=4). Vertical dashed lines indicate the plate-specific transitions between baseline, cell seeding/growth, treatment, and recovery phases. These transitions occurred at 18, 44, and 65 h for CAL-33 and at 24, 49, and 73 h for hASC. Extract 16 induced sustained impedance suppression relative to the DMSO control in CAL-33 cells, followed by partial late recovery. In contrast, hASC did not exhibit a comparable treatment-associated impedance suppression. The frequency-dependent profiles provide additional biophysical resolution of the cell-type-specific response but do not independently establish a specific molecular or structural mechanism.

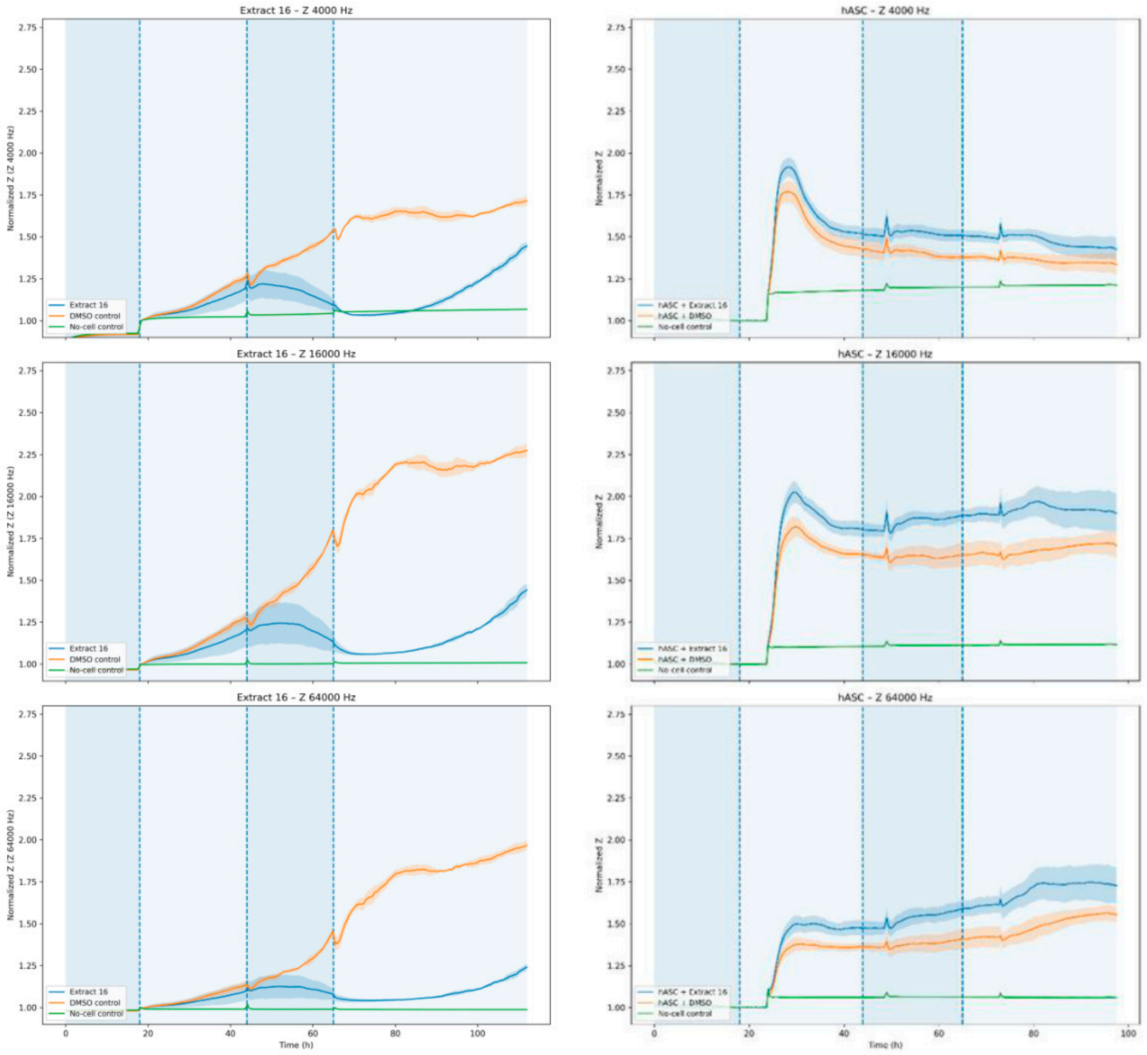

Supplementary Figure S4. Treatment-associated redistribution of β-catenin and ZO-1 in Detroit 562 cells. Representative confocal images showing β-catenin and ZO- 1 localization in Detroit 562 cells following treatment with DMSO, Extract 16, Inavolisib, or the combination treatment (Combo). Treatment was associated with changes in cellular morphology and spatial redistribution of β-catenin and ZO-1 compared with the DMSO control. The observed patterns indicate altered junctional organization rather than a uniform loss of protein immunoreactivity. Nuclei were counterstained with DAPI. Imaging settings were kept constant within each marker series. Scale bars are indicated in the individual images. Representative images from two independent biological experiments (n=2) are shown. Treatment concentrations and incubation periods are described in the Materials and Methods section.

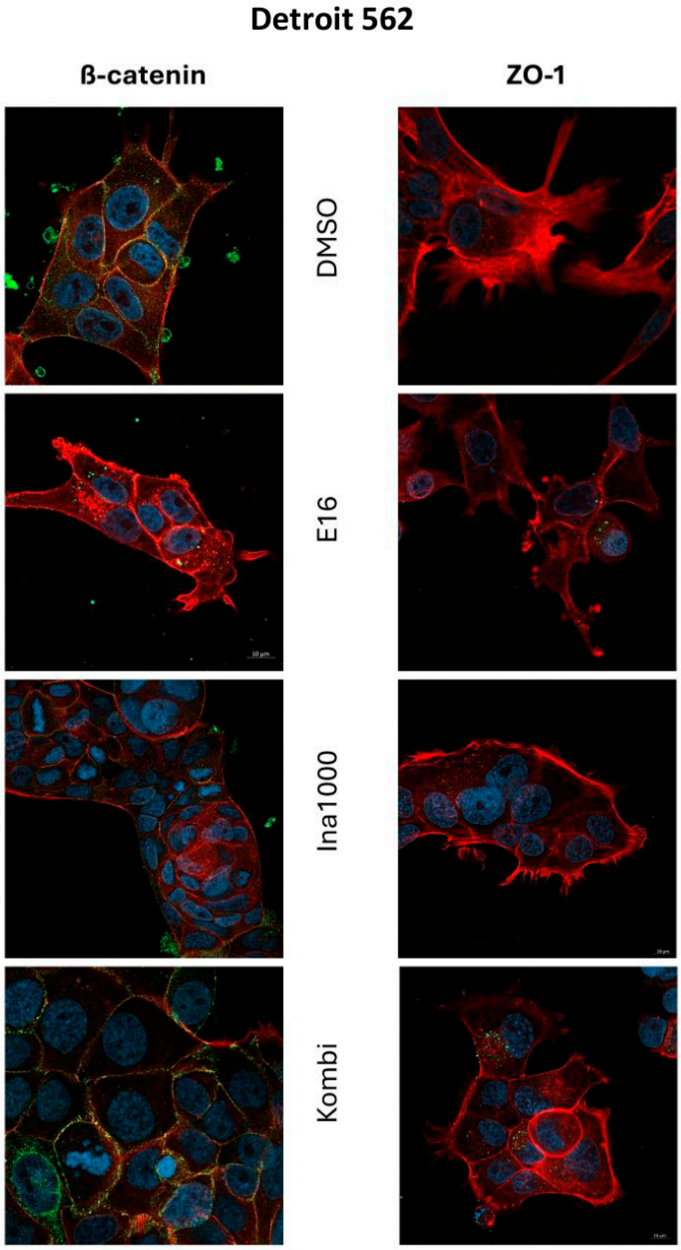

Supplementary Figure S5. No treatment-associated increase in caspase 3 immunoreactivity in CAL-33 and Detroit 562 cells. Representative confocal images showing caspase 3 immunoreactivity in CAL-33 and Detroit 562 cells following treatment with DMSO, Extract 16, or the combination treatment (Combo). Caspase 3 is shown in red, and nuclei were counterstained with DAPI. No consistent treatment-associated increase in caspase 3 immunoreactivity was observed in either cell line. Because cell density and the number of remaining cells differed between treatment conditions, the images are interpreted qualitatively and do not provide a quantitative comparison of total fluorescence intensity. Imaging settings were kept constant within each cell-line series. Scale bars are indicated in the individual images. Representative images from two independent biological experiments (n=2) are shown..

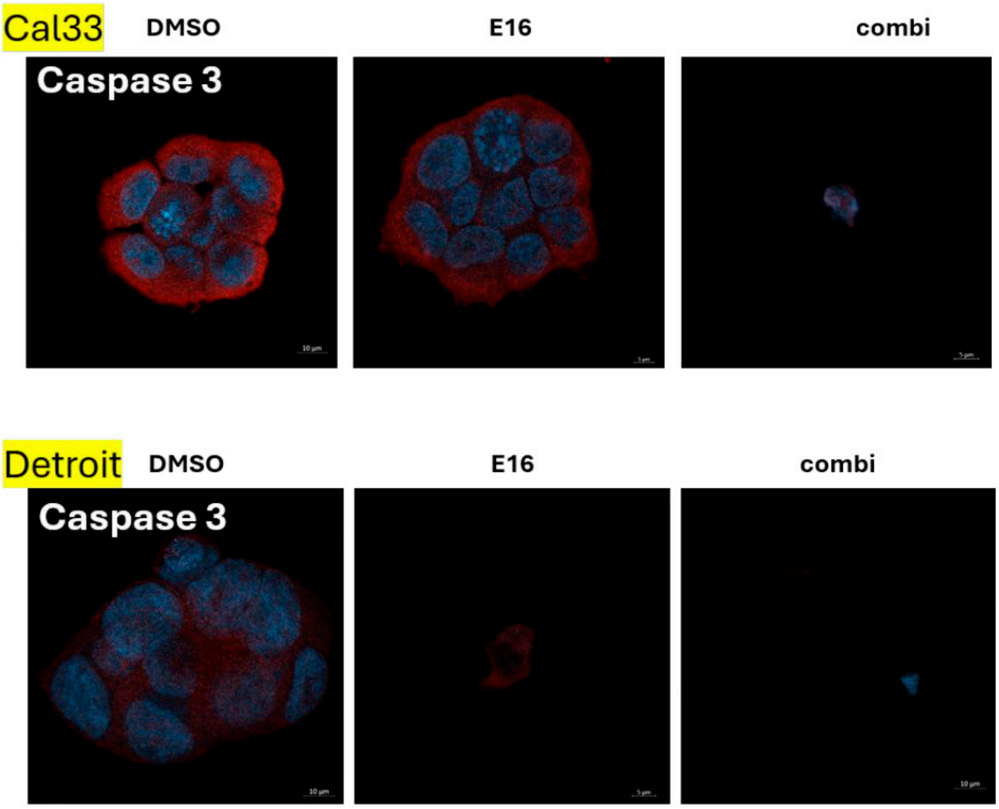

Supplementary Figure S6. Original immunoblot and stain-free total-protein images. Original immunoblot images corresponding to the analysis of total PARP, cleaved PARP, phosphorylated AKT (p-AKT), phosphorylated mTOR (p-mTOR), and β-actin in CAL-33 and Detroit 562 cells following treatment with DMSO, Extract 16 (E16), Inavolisib (Ina), or the combination treatment (Combo).. Stain-free total-protein images corresponding to the respective membranes are shown in panels A–C and were used to control protein loading and transfer and for densitometric normalization. β-Actin is shown as an additional loading-control marker but was not used as the primary normalization reference. The images shown are representative of two independent biological experiments (n=2). Complete original images from both experiments, including molecular-weight markers and membrane boundaries, are provided in the accompanying source data.

Original western blots

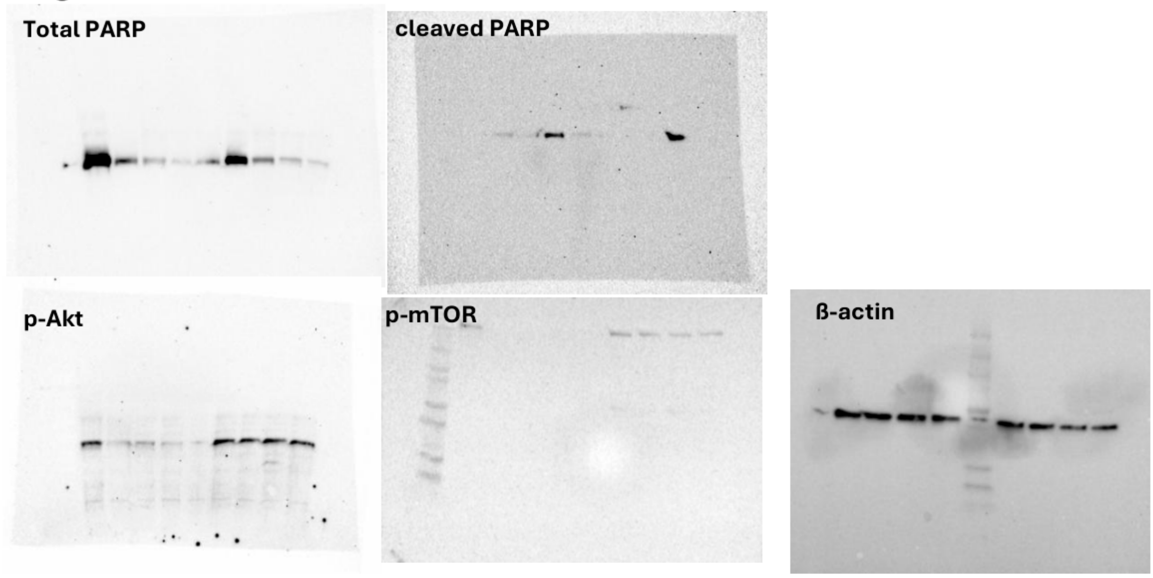

Stain-free images

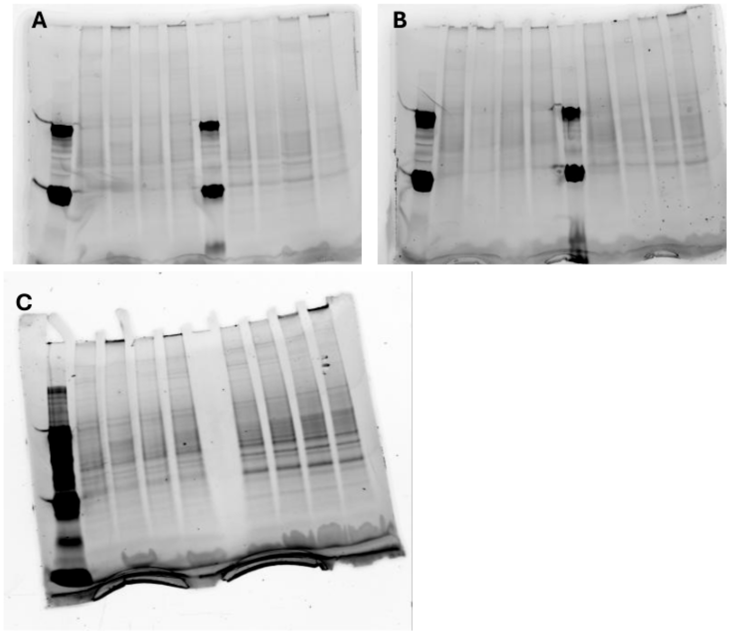

Supplementary Figure S7. Exploratory comparison of CAL-33 metabolic responses at different levels of cell confluence. Metabolic activity was assessed in CAL-33 cells treated for 24 h with DMSO, Inavolisib (Ina; 1000 nM), Extract 16 (E16; 50 µg/mL), or their combination (Combo; 1000 nM Inavolisib plus 50 µg/mL Extract 16). Cells were treated at approximately 70% confluence in the first experiment and at approximately 90–100% confluence in the second experiment. MTS signals were normalized to the corresponding DMSO control, which was defined as 100%. Boxplots and individual values represent six technical replicates for the approximately 70% confluent experiment (n=6) and seven technical replicates for the approximately 90–100% confluent experiment (n=7). Extract 16 alone increased the normalized metabolic signal above the DMSO control under both conditions, whereas Inavolisib reduced metabolic activity. The relative response to Combo differed between the two experiments: Combo produced a stronger reduction than Inavolisib alone at approximately 70% confluence but a weaker reduction than Inavolisib alone at approximately 90–100% confluence. Because the two confluence conditions were investigated in separate representative experiments and the data comprise technical replicates, the comparison is exploratory and descriptive. The results indicate that cell density or cell state may influence the measured pharmacological response and emphasize the need for standardized confluence in comparative MTS experiments.

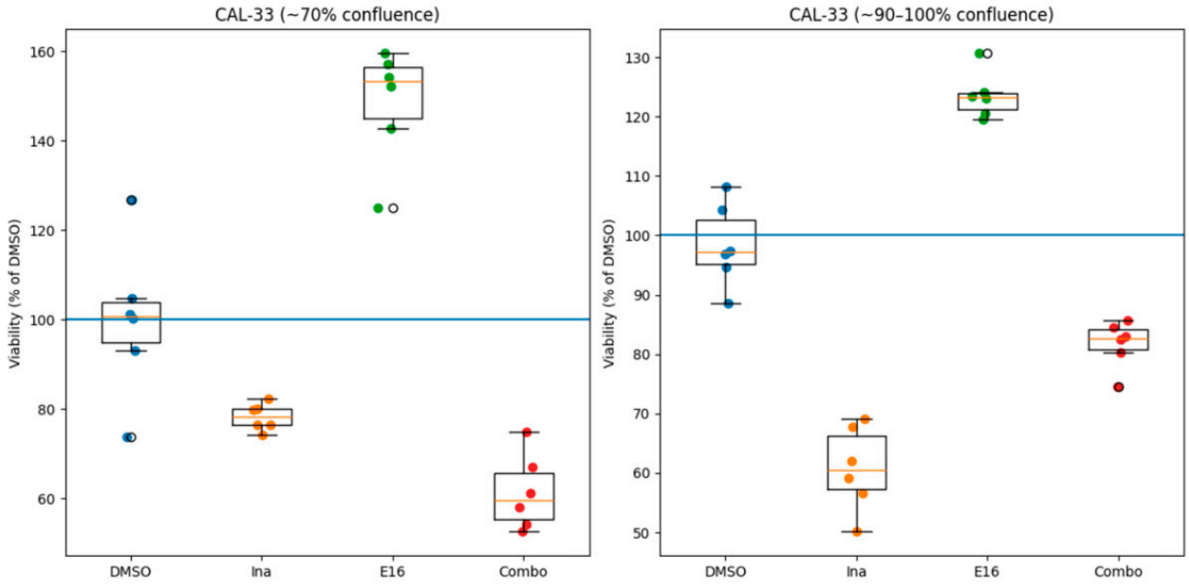

Supplement: Supplementary file 1 [file biosensors-16-00563-s001.zip › biosensors-4539069-supplementary.pdf]
